# Modelling Tetralogy of Fallot: insights and limitations of animal models

**DOI:** 10.3389/fcell.2026.1889136

**Published:** 2026-08-17

**Authors:** Dimitra Mouzourou, Haoxiang Zhou, Bernard Keavney, Kathryn E. Hentges

**Affiliations:** 1 Division of Evolution, Infection and Genomics, BHF Manchester Centre of Research Excellence, School of Biological Sciences, Faculty of Biology, Medicine, and Health, Manchester Academic Health Science Centre, University of Manchester, Manchester, United Kingdom; 2 Division of Cardiovascular Sciences, Faculty of Medicine, Biology and Health, Manchester Centre of Research Excellence, University of Manchester, Manchester, United Kingdom; 3 BHF Manchester Centre of Research Excellence, University of Manchester, Manchester, United Kingdom

**Keywords:** animal models, cardiac septation, congenital heart disease, genetics, outflow tract, tetralogy of fallot

## Abstract

Tetralogy of Fallot (TOF) is the commonest cyanotic congenital heart disease, arising from antero-cephalad deviation of the outlet septum during outflow tract (OFT) morphogenesis, producing a ventricular septal defect, overriding aorta, pulmonary stenosis, and right ventricular hypertrophy. Despite advances in surgical correction, the developmental origins of TOF remain incompletely understood, and the genetic architecture of the majority of non-syndromic cases is yet to be resolved. Experimental models have been central to progress in this field, yet each model may capture only part of the disease. This review evaluates animal and genetic models of TOF across species, discussed in order of increasing cardiovascular similarity to humans. Zebrafish and *Xenopus* enable rapid *in vivo* interrogation of candidate genes and conserved developmental pathways, despite fundamental differences in cardiac anatomy. Avian models have been instrumental in defining the contributions of the second heart field and cardiac neural crest cells to OFT elongation and septation. Mouse models, with their four-chambered heart and amenability to precise genetic modification, have provided the most detailed mechanistic insights, and we examine key models across the multiple signalling pathways in depth. A recurring finding is that individual models reproduce discrete components of the TOF tetrad rather than its complete set of defects, frequently producing double outlet right ventricle or persistent truncus arteriosus rather than classical TOF. No single model fully recapitulates all four features with complete penetrance. Continued integration of human genomic data with targeted experimental perturbations will be essential for closing this gap.

## 1 Introduction

Tetralogy of Fallot (TOF) is a form of congenital heart disease (CHD) that has been recognised for over three centuries (Neill and Clark, 1994). TOF is the commonest cyanotic congenital heart condition, occurring in approximately 3 per 10,000 live births and accounting for roughly 5%–10% of all CHD cases worldwide (Bailliard and Anderson, 2009). Its prevalence and severe clinical presentation have made TOF a focal point in congenital cardiovascular research, driving major advances in cardiac surgery and developmental cardiology. Prior to surgical intervention, only 24% of children with TOF lived to 10 years of age, however, today, TOF patients who are surgically treated survive into middle-to-late adulthood, though lifelong monitoring is required due to residual cardiac dysfunction (Bertranou et al., 1978). The historical significance, prevalence and etiological complexity of TOF have driven the development of diverse experimental models aimed at recapitulating its anatomical, molecular and functional features.

### 1.1 Anatomical features and clinical significance

**FIGURE 1:**
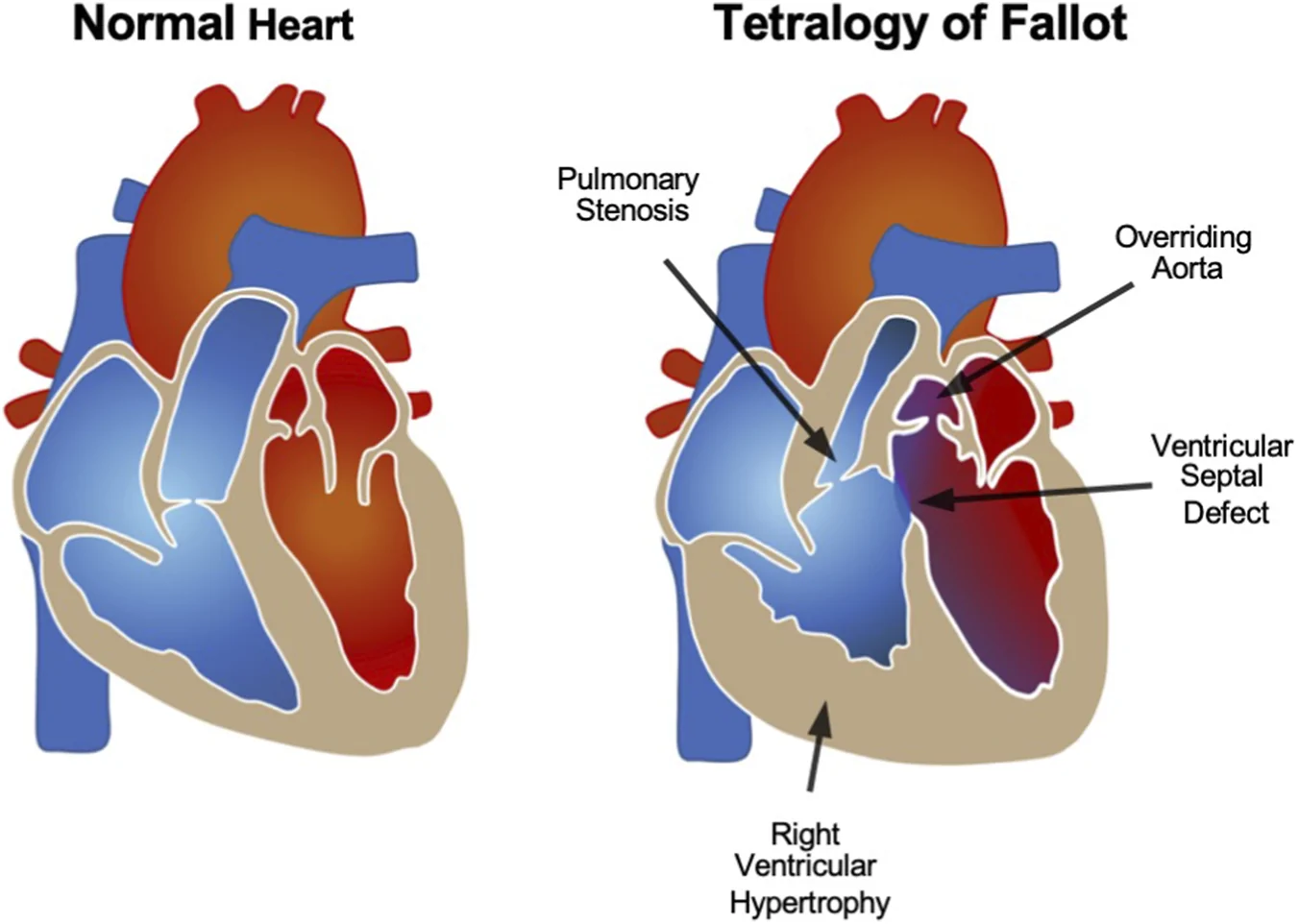
Schematic representation of a normal heart and the altered cardiac anatomy of Tetralogy of Fallot. TOF is comprised of four defining anatomical defects: *Overriding aorta* (OA), where the aortic outlet straddles the ventricular septum and receives blood from both ventricles, *Right ventricular outflow tract (OFT) obstruction usually caused by pulmonary stenosis, Ventricular septal defect (VSD)*, typically a single malalignment opening in the outlet septum, and *Right ventricular hypertrophy*, a secondary consequence of pressure overload from the outflow obstruction.

In humans, the defects contributing to TOF arise during outflow tract (OFT) and conotruncal septation in early cardiac embryogenesis, between approximately 4–8 weeks of gestation (Lamers and Moorman, 2002). The four cardiac defects presented in TOF include ventricular septal defect (VSD), stenosis of the pulmonary trunk due to OFT obstruction, an overriding (dextroposed) aorta, and lastly enlargement of the right ventricle, termed right ventricular hypertrophy (Figure 1).

During embryogenesis, the cardiac neural crest cells (cNCCs) and secondary heart field (SHF) progenitors orchestrate the elongation and division of the OFT into the aorta and pulmonary arteries (Stefanovic et al., 2021). An antero-cephalad deviation of the outlet septum, often accompanied by hypertrophy of septoparietal trabeculations, underlies the characteristic lesions of TOF. Malalignment of the outlet septum produces a large, non-restrictive VSD and an overriding aorta, in which the aortic root straddles the interventricular septum and receives blood from both ventricles (Bailliard and Anderson, 2009). When septal malalignment is combined with infundibular (subvalvular) narrowing of the subpulmonary OFT, a right ventricular outflow tract (RVOT) obstruction develops. This chronic pressure overload leads to right ventricular hypertrophy, completing the anatomical tetrad (Geva et al., 2024).

RVOT obstruction may occur at multiple levels including infundibular, valvar, or branch pulmonary arteries, and its severity is the major determinant of clinical outcome (Starr, 2010). Increased resistance to pulmonary blood flow elevates right ventricular pressure above that of the left ventricle, producing a right-to-left shunt across the VSD. The mixture of deoxygenated blood into the systemic circulation causes cyanosis, the hallmark of TOF. The right ventricle adapts to this pressure load through compensatory hypertrophy (Alipour et al., 2023).

Clinically, TOF spans a spectrum. In acyanotic TOF, mild obstruction allows predominantly left-to-right shunting and near-normal oxygenation. Conversely, severe obstruction or VSD with pulmonary atresia (TOF/PA) results in profound cyanosis from birth (Ganigara et al., 2021). In preoperative infants and incompletely palliated adults with TOF, the dynamic nature of the infundibular obstruction can trigger hypercyanotic “Tet” spells, acute episodes of diminished pulmonary blood flow and systemic desaturation (Bailliard and Anderson, 2009). These hemodynamic consequences highlight why RVOT obstruction is regarded as the critical determinant of disease severity.

Although complete surgical correction is now routine in infancy, long-term complications including pulmonary regurgitation, arrythmias, and sudden cardiac death may persist (Kauling et al., 2025). Thus, the interplay between structural malalignment, variable outflow obstruction, and secondary hypertrophy explains both the anatomical complexity and clinical heterogeneity of TOF. Understanding these mechanisms provides a foundation for evaluating experimental models, which aim to replicate the developmental origins and pathophysiological features of this cyanotic CHD.

### 1.2 Genetic underpinnings

Genetically, TOF is heterogeneous, involving both chromosomal syndromes and numerous genetic mutations that disrupt cardiac developmental pathways. Approximately 20% of TOF cases occur in the context of defined syndromes or chromosomal abnormalities (Althali and Hentges, 2022). Classic examples include aneuploidies such as Down syndrome (trisomy 21) and Turner syndrome (45,X), and microdeletions with high CHD penetrance including DiGeorge syndrome (22q11.2 deletion) (Guo et al., 2011). TOF can also be caused by single-gene disorders, including Alagille syndrome caused by deletions or variants within JAG1 (94%–97% of cases) and NOTCH2 (1%–3% of cases) (Althali and Hentges, 2022). The majority (80%) of TOF cases are non-syndromic (isolated) with no obvious cytogenetic causes (Althali and Hentges, 2022). Research over the past two decades has identified a growing list of gene variants underlying these non-syndromic cases. Deleterious mutations in *NKX2.5*, *GATA4*, *TBX5*, *JAG1 (Jagged1), ZFPM2 (FOG2*), and other transcriptional regulators required for heart tube expansion and septation have been found in sporadic TOF cases (Althali and Hentges, 2022). These genes converge on critical pathways that pattern the right ventricular outflow region, for instance *TBX1* from the SHF and *Jagged/Notch* signalling in neural crest cells both influence outflow tract separation (Stefanovic et al., 2021; High et al., 2007).

## 2 Scope of this review

Given the anatomical complexities of TOF, there is a pressing need for diverse experimental approaches, from non-mammalian vertebrate models to genetically engineered mice, to better elucidate TOF pathogenesis. The structure of this review was informed by current concepts of TOF and DORV as part of a broader spectrum of OFT alignment defects resulting from perturbations in OFT elongation, rotation, septation and remodelling (Kelly, 2024). We identified animal and genetic models of TOF through PubMed, Web of Science, and Scopus using combinations of search terms including Tetralogy of Fallot, conotruncal, outflow tract, together with the species and gene names discussed, covering literature published up to September 2025. We focused on models in which genetic or surgical perturbation produced outflow tract malalignment relevant to TOF, across zebrafish, *Xenopus*, chick, and mouse. Where multiple animal models or genetic alterations were generated for the same gene, we preferentially presented those that most closely recapitulated the cardinal features of TOF.

This review concentrates on animal and genetic models of TOF, rather than on its human genetic architecture or its non-genetic causes. The genetic variants associated with human TOF (Althali and Hentges, 2022) have been reviewed in detail elsewhere, as have epigenetic and environmental contributors, including the maternal–foetal microRNA axis (Calcaterra et al., 2026). These aspects therefore lie outside the scope of the present review, and we direct readers to those publications.

In this review, the models are discussed in order of increasing anatomical and physiological similarity to the human heart, from zebrafish to mouse, highlighting the complementary advantages of each model for investigating different aspects of TOF. We examine genetic models, with a particular focus on engineered mouse mutants that highlight key genes and molecular pathways implicated in TOF. By correlating findings from these complementary systems, we aim to provide a broad summary of the developmental mechanisms underlying TOF. Ultimately, although no single experimental model completely recapitulates the anatomical and genetic complexity of human TOF, insights gained from these anatomical, surgical and genetic approaches continue to advance our understanding of the developmental origins of TOF, and may in future inform therapeutic or preventative strategies.

## 3 Animal models across different species

**FIGURE 2:**
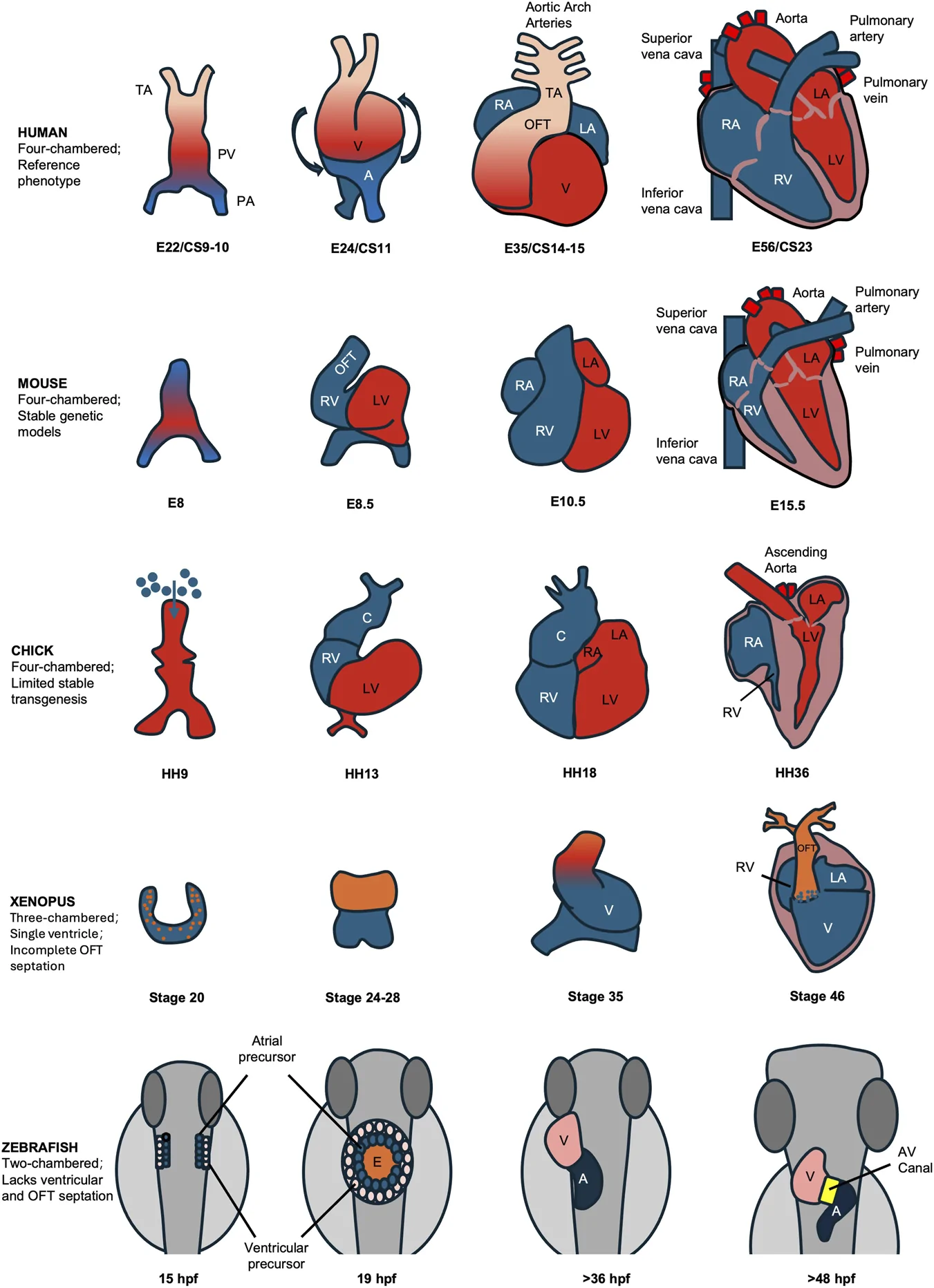
Stages of heart development in human, mouse, chick, *Xenopus* and zebrafish in a ventral view. The panels illustrate the progressive morphogenesis of the early linear tube into a chambered heart, highlighting conserved and species-specific features of cardiac development. In human and mouse, looping of the primitive heart tube and outflow tract is accompanied by septation of the atria and ventricles, remodelling of the aortic arch arteries, and formation of the pulmonary artery, pulmonary veins and venae cavae. In chick and *Xenopus*, the early tubular heart undergoes looping and regionalisation to generate distinct atrial and ventricular chambers, culminating in the establishment of anatomically separate inflow and outflow tracts. In zebrafish, the emergence of atrial and ventricular precursors (cardiomyocytes), subsequent development of the atrioventricular (AV) canal are depicted across successive hours post fertilisation (hpf). These anatomical differences determine which aspects of TOF each system can model. The four-chambered, septated hearts of mouse and human support the full outflow tract and septation defects of TOF, whereas the four-chambered chick heart is suited to surgical and morphogenetic manipulation of OFT development. The single-ventricle hearts of *Xenopus* and zebrafish, which lack ventricular septation (and, in *Xenopus*, neural crest-dependent OFT septation), cannot reproduce the complete structural lesions of TOF but allow rapid testing of conserved gene function. E = embryonic day; CS = Carnegie Stage; HH = Hamburger Hamilton stage; TA = Truncus arteriosus; PV = primitive ventricle; PA = primitive atrium; A = atrium; AV = atrioventricular; C = conus; E = endocardium; LA = left atrium; LV = left ventricle; OFT = outflow tract; RA = right atrium; RV =right ventricle; V = ventricle.

To better understand the developmental mechanisms underlying CHD, including TOF, a wide range of animal models has been used over the past several decades. Zebrafish, *Xenopus* and chick embryos are all vertebrate models that provide experimentally accessible systems for studying cardiac development, despite important differences from mammalian heart structure. Zebrafish possess a two-chambered heart, *Xenopus* develops a three-chambered heart, and the chick heart is four-chambered and shares many aspects of cardiac morphogenesis with mammals (Figure 2). Mouse models provide the closest approximation to human cardiovascular development among commonly used experimental systems. The following sections discuss each model system in turn, highlighting their respective advantages, limitations and applications in the study of TOF.

### 3.1 Zebrafish models

Zebrafish offer a low-cost, high-throughput genetic model for studying cardiac development. Their external fertilisation, rapid embryonic development, optical transparency and high fecundity make zebrafish particularly amenable to large-scale genetic screening. Combined with the fact that approximately 70% of human protein-coding genes have at least one zebrafish orthologue (Howe et al., 2013), the advantages of the zebrafish model enabled large-scale ENU mutagenesis screens that identified numerous genes involved in vertebrate cardiac development and established zebrafish as a major model for cardiovascular research (Stainier et al., 1996). Despite these advantages, zebrafish have significant limitations for modelling the anatomical features of TOF. The zebrafish heart consists of a single atrium and single ventricle and lacks ventricular septation (Figure 2), preventing the faithful recapitulation of defects such as VSD and overriding aorta. Furthermore, the zebrafish OFT contains a bulbus arteriosus that differs substantially from the mammalian OFT, and does not reproduce the complex septation and valvular structures involved in human TOF. Nevertheless, zebrafish remain highly valuable for candidate gene screening and functional validation. Cardiac developmental abnormalities such as looping defects and cardia bifida are readily identifiable, and early embryos can survive for several days despite severe cardiovascular dysfunction owing to oxygen diffusion, facilitating detailed phenotypic analysis. Techniques such as morpholino knockdown, mRNA overexpression, CRISPR-based mutagenesis and rescue experiments therefore allow rapid assessment of gene function during cardiac development.

For example, a zebrafish morpholino knockdown model of blood vessel epicardial substance (BVES) exhibited pronounced looping defects and stenosis of the ventricular outflow tract (VOT), accompanied by reduced expression of the transcription factors *Nkx2.5* and *Gata4* in the SHF (Shi et al., 2020). These molecular alterations resemble perturbations reported in patients carrying BVES mutations (Wu et al., 2013). As such, zebrafish are particularly useful for identifying and prioritising genes involved in CHD and TOF pathogenesis, although confirmation of disease mechanisms and anatomical phenotypes generally requires more complex vertebrate models.

### 3.2 *Xenopus* models

The *Xenopus* model is widely used in developmental biology and cardiovascular research. Its large externally developing embryos, ease of microinjection, and well-characterised embryogenesis make *Xenopus* particularly suitable for rapid gain- and loss-of-function studies during early cardiac development.

However, *Xenopus* has important limitations as a model of TOF. The *Xenopus* heart consists of two atria and a single ventricle, and its OFT remains incompletely septated (Figure 2). Furthermore, species-specific differences in cardiac development such as a lack of neural crest cell contribution to OFT septation, *Xenopus* cannot fully recapitulate the anatomical complexity of human TOF (Lee and Saint-Jeannet, 2011).

Despite these limitations, *Xenopus* has proven valuable in dissecting the genetic basis of CHD and conotruncal malformations through functional perturbation of candidate genes identified in human studies. Expression of *NKX2.5* mutations identified in human patients, including those with TOF, causes ASD and abnormal valve formation in *Xenopus*, mirroring the cardiac manifestations observed in those patients (Bartlett et al., 2007). In models of *chd7* deficiency, *Xenopus* embryos exhibit impaired neural crest migration, partially recapitulating the cardiovascular presentation of CHARGE syndrome patients, including TOF (Bajpai et al., 2010).

Consequently, similar to zebrafish, *Xenopus* serves as a useful platform for validating candidate genes and developmental pathways implicated in CHD and TOF, enabling rapid assessment of the functional consequences of variants identified in human genetic studies.

### 3.3 Avian (chick) models

Chick embryos provide a classic surgical model of TOF. Like humans, the avian heart develops into four chambers and a septated outflow tract, making chick embryos particularly valuable for modelling conotruncal defects such as TOF, despite species-specific differences in timing and scale of morphogenesis (Figure 2). One of the first successful chick TOF models was created in 1985, by placing a silk filament around the conoventricular groove of embryos at Hamburger-Hamilton (HH) stages 17–21 (Aranega et al., 1985). This mechanical interference was thought to delay normal regression of the groove and impair OFT remodelling. Of the 397 operated embryos, only 26 survived to HH35, of which 22 presented cardiac malformations. Of these, 86% had pulmonary stenosis, and 50% showed a combination of cardiac anomalies such as OA, pulmonary stenosis, and VSD, similar to TOF in humans (Aranega et al., 1985). Two decades later, a different surgical approach involving ablation of the SHF at HH14 produced comparable results, as 76% of operated chick embryos developed cardiac defects, including pulmonary stenosis, pulmonary atresia and OA (Wa et al., 2005). Additionally, several embryos demonstrated displacement of the septum, VSD, and in severe cases coronary artery anomalies (CAAs) (Wa et al., 2005).

The Aranega et al. (1985) model demonstrated that mechanical interference with the conoventricular groove could reliably produce TOF-like anatomy, laying the groundwork for more targeted approaches (Aranega et al., 1985). Subsequent ablation of the SHF at HH14 by Ward et al. revealed that SHF-derived cells contribute to the distal myocardium, promoting elongation and proper rotation of the OFT (Wa et al., 2005). Consistent with this, chick neural crest ablation, which secondarily prevents SHF-derived myocardium from entering the OFT, results in persistent TA, DORV, or TOF anatomy (Wa et al., 2005). These findings highlight that insufficient extension of the OFT by SHF-derived myocardium is a key developmental mechanism underlying misalignment of the great arteries, VSD, and OFT obstruction that define TOF in humans.

Today, although chick embryos are not typically used to establish the genetic basis of CHD, in part because stable germline genetic manipulation is less well established than in mouse and zebrafish models (Intarapat and Stern, 2013), they remain well suited to analysing the cellular and morphogenetic mechanisms underlying OFT development, owing to their accessibility for spatially and temporally controlled experimental manipulation (Stern, 2005).

### 3.4 Mouse models

The laboratory mouse remains the cornerstone for genetic studies of conotruncal heart defects and CHD more broadly, because its four-chambered heart closely mirrors human cardiac anatomy (Figure 2), and stable germline genetic modification can be readily achieved. This approach allows direct assessment of how specific genetic alterations influence cardiac development and lead to structural defects. Numerous engineered mouse lines exhibit conotruncal malformations, including abnormalities in OFT alignment, septation, and ventricular septal formation (Table 1). However, these phenotypes frequently fall within a broader spectrum of OFT malalignment, and in some instances more closely resemble variants of double outlet right ventricle (DORV) rather than classical human TOF (Table 1) (Kelly, 2024). Although early embryonic lethality in some mutants and species-specific differences can complicate interpretation (Table 1), the mouse remains an indispensable system for dissecting the genetic basis of TOF and related conotruncal defects.

**TABLE 1:** Mouse models with TOF-like cardiac phenotypes, with penetrance, sample size, embryonic stage, concurrence of the four-feature tetrad within a single animal, and the principal limitation of each model. Asterisks (*) mark entries requiring qualification.

Model (pathway)	TOF components present VSD | OA | PS/PA | RVH	Penetrance of key lesions	Stage(s) examined	Full tetrad in same animal?	Other anomalies	Lethality	Notes	Reference
*Hey2 ^−/−^* (Notch)	✓ | ✓ | ∼ | ∼	VSD + OA in 100% of embryos examined (n > 20); PS/OFT obstruction in ∼40% (n = 15); postnatal RVH	E14.5-E15.5 P0-P1 Weaning stage	Partial. VSD+OA+PS co-occur in ∼40%; RVH seen postnatally → Full tetrad frequency not reported	Tricuspid atresia ASD	Early postnatal lethality	Postnatal lethality limits RVH documentation; penetrance background-strain dependent	Donovan et al. (2002)
*Notch1^+/−^; Nos3^−/−^* (Notch)	∼ | ∼ | ✓* | NR	VSD (33%), OA (33%), *thickened pulmonary valve (88%); (n = 9); no RVH but enlarged RV	E11.5–18.5 P10	Partial. VSD+OA+PS frequency not reported	BAV; thickened semilunar valves (stenosis); Enlarged RV	Embryonic/perinatal (∼65% by P10)	∼65% lethality by P10 prevents RVH; compound (two-gene) genotype; Valve lesions extend beyond TOF; *PS not stated	Koenig et al. (2016)
*VEGF^120/120^* (VEGF–Notch)	∼ | ∼ | ∼ | NR*	VSD+ OA+ PS in 29% of embryos examined (n = 31); subset progress to pulmonary atresia	E10.5-E19.5; perinatal	Partial. VSD+ OA+ PS co-occur in 29% → *TOF reported but full tetrad not exhibited	Thin myocardium; diminished looping; right/double aortic arch	Perinatal lethality	Isoform model; uniquely captures TOF/PA; *TOF not defined (RVH not mentioned)	van den Akker et al. (2007), Rammeloo et al. (2015)
*Jagged1^ECKO^*	✓ | ✓ | ∼ | ✓	OA (30%; n = 46) VSD; RVH; PS; (not quantified)	E10.5 Neonatal Adult	Partial. VSD + OA+ RVH documented together; frequency not reported; PS from pulmonic valve stenosis	Adult valve calcification (5/10); hypoplastic cushions	Variable (mosaic Cre); survive to adulthood	Penetrance variable due to mosaic VE-Cadherin-Cre; PS inferred from valve stenosis; models Alagille syndrome; valve calcification in addition to TOF tetrad	Hofmann et al. (2012)
*Robo1^I270T/I270T^* (SLIT–ROBO; ENU)	✓ | ✓ | NR | NR*	VSD, OA, (Not quantified) *No RVH but biventricular hypertrophy	E18.5	Partial. VSD+OA+ biventricular hypertrophy; frequency not reported	ASD; AVSD; DORV; cleft palate; micrognathia	NR	No survival data; PS and RVH not reported; ASD/AVSD and craniofacial defects in addition to TOF	Kruszka et al. (2017)
*Cited2^−/−^*	✓ | ✓ | NR | NR*	VSD + OA in (84%) of embryos examined (n = 19) *No RVH but ventricular hypertrophy (not quantified)	E10.5–E17.5	Partial. VSD+OA+ ventricular hypertrophy; frequency not reported	ASD; dilated RV; exencephaly; pulmonic arterial stenosis	Embryonic (mid-gestation; some survive later)	Variable lethality; pleiotropic (laterality, exencephaly, adrenal defects); TOF tetrad not concurrent	Bamforth et al. (2001), Yin et al. (2002)
*Fog2^−/−^*	✓ | ✓ | ✓ | NR	VSD, OA, PS (Not quantified)	E12.5-E15	Partial. VSD+OA+PS frequency not reported	Common AV canal; contiguous ASD+VSD; thin myocardium	Embryonic (E12.5–E14.5)	Embryonic lethality precludes RVH detection; common AV canal/ASD in addition to TOF	Tevosian et al. (2000)
*Tbx1^neo2/neo^* (hypomorph, ∼20% mRNA)	✓ | NR | NR | NR	VSD (85%, n = 13)	E9.5-E18.5	VSD → *TOF reported but full tetrad not exhibited	DORV; TAC; thymic aplasia	100% neonatal lethality	OFT scored as VSD/DORV/TAC; *TOF not defined (PS, OA, RVH not mentioned)	Zhang and Baldini (2008), Majumdar et al. (2021)
*Shh* ^−/−^	✓ | ✓ |✓ | ∼	VSD + OA+ PA (100%, n = 5); variable ventricular hypertrophy	E8-E18.5	Yes. VSD + OA+ PA+ ventricular hypertrophy variable → Frequency not reported	ASD; absent ductus arteriosus; pharyngeal and craniofacial defects	Perinatal (die at birth)	PA atresia from failed 4th/6th arch artery formation + caudal pharyngeal hypoplasia, no septation failure; hypertrophy likely secondary; severe pleiotropy	Smoak et al. (2005)
*Mef2c* ^AHF−KO^	✓ | ∼ | ∼ | ✓	VSD + OA+ RVH + stenotic/atretic PA (14%, n = 36)	E10.5-E12.5 P0	Yes. All four concurrently, including RVH (14%, n = 36)	TGA; DORV; thickened semilunar valves; shortened aorta	∼50% neonatal	Alignment-defect spectrum (TOF/DORV/d-TGA) from one lesion; TOF a subset; mixed background modifies penetrance	Barnes et al. (2016)
*Lrp6^−/−^* (canonical Wnt)	✓ | NR | NR | NR	VSD (Not quantified)	E9.5-E16.5	No. VSD + DORV (override)	DORV; PTA	Most survive to birth; some die E9.5–E11.5	PS not clearly observed; phenotype is TGA/DORV override rather than TOF	Song et al. (2010), Bryja et al. (2009)
*Ctnnb1^Dermo1−Cre^* (canonical Wnt, mesoderm)	✓ | ✓ | NR | NR	VSD (100%) + OA (53%) in embryos examined (n = 15)	E9.5-E17.5	No. VSD + OA.	DORV; PTA; ASD; pharyngeal arch artery anomalies; hypoplastic PA branches	NR	RVH not observed; PS is branch-PA hypoplasia, not valvar/infundibular	Huh and Ornitz (2010)
*Wnt5a^−/−^* (non-canonical Wnt/PCP)	✓ | NR | NR | NR	VSD (100%, n = 19);	E9.5–E17.5	No. VSD + OFT malformations (100%); predominant phenotype is PTA	PTA; DORV; TGA; IAA	Neonatal lethality	Predominant phenotype is PTA; perinatal lethality	Schleiffarth et al. (2007), Sinha et al. (2015)
*Wnt11^−/−^* (non-canonical Wnt)	✓ | NR | NR | NR	VSD (Not quantified)	E9.5-E14.5	No. VSD + OFT malformations	ASD; TGA; DORV; PTA	Embryonic and perinatal lethality	Phenotype is DORV/TGA malalignment; myocardial thinning	Nagy et al. (2010), Zhou et al. (2007), van Vliet et al. (2017)
*Vangl2^Lp/Lp^* (non-canonical Wnt/PCP)	✓ | NR | NR | NR	VSD (100%, n = 18)	E8.5-E18.5	No. VSD + DORV (100%, n = 18) phenotype	DORV; right/double aortic arch; abnormal heart looping; craniorachischisis	Perinatal lethality driven by craniorachischisis	DORV not TOF; severe NTD pleiotropy; OFT defect may arise from neuroepithelium not cNCC	Henderson et al. (2001), Ramsbottom et al. (2014)
*Pkhd1l1^−/−^* (ciliary; oligogenic screen)	∼ | ∼ | ∼ | NR	VSD + OA (12%, n = 73); PA (Not quantified)	E10.5-E13.5 P1	Partial. OA+VSD+PA; frequency not reported	ASD; thinner pulmonary arteries; OFT/aorta–PA malrotation	Postnatal lethality (8.22% by P1)	Single-gene effect modest (oligogenic context needed); incomplete penetrance	Zhou et al. (2025)

Tetrad key: ✓ reported/consistently present; ∼ present in a subset or low penetrance; NR, not reported; ASD, atrial septal defect; AV, atrioventricular; AVSD, atrioventricular septal defect; BAV, bicuspid aortic valve; cNCC, cardiac neural crest cells; DORV, double-outlet right ventricle; E, embryonic day; ENU, *N*-ethyl-*N*-nitrosourea; IAA, interrupted aortic arch; mRNA, messenger RNA; NR, not reported; NTD, neural tube defect; OA, overriding aorta; OFT, outflow tract; P, postnatal day; PA, pulmonary atresia; PCP, planar cell polarity; PS, pulmonary stenosis; PTA, persistent truncus arteriosus; RV, right ventricle; RVH, right-ventricular hypertrophy; TAC, truncus arteriosus communis; TGA, transposition of the great arteries; TOF, tetralogy of Fallot; VEGF, vascular endothelial growth factor; VSD, ventricular septal defect.

Throughout this review, we reserve the term Tetralogy of Fallot for models reproducing at least three of the four core features: a ventricular septal defect, overriding aorta, pulmonary stenosis or atresia, and right ventricular hypertrophy. Models in which the predominant lesion is double outlet right ventricle, persistent truncus arteriosus or transposition of the great arteries are described as TOF-like or conotruncal alignment defects, rather than as complete TOF models. Phenotypes are otherwise reported using the classifications applied in the original publications.

#### 3.4.1 Notch family

The *Notch* signalling pathway plays a well-established role in the pathogenesis of TOF and related conotruncal heart defects. Early research showed that Notch signalling coordinates a wide array of cellular processes essential for cardiac development, particularly through its influence on the neural crest cell (NCCs) population (High et al., 2007; Loomes et al., 2002) and endothelial-to-mesenchymal transition (EndMT) (Timmerman et al., 2004). In mice, around embryonic day E8.5, a specialised group of NCCs that originate from the dorsal neural tube, the cardiac neural crest cells (cNCCs), begin to migrate through the pharyngeal arches and into the truncus arteriosus (TA) (High et al., 2007). These cells contribute to the formation of the OFT septum, and the aortopulmonary cushions (Kirby et al., 1983). Concurrently, endocardial cells regulated by *Notch* signalling undergo EndMT, a process critical for cardiac cushion formation, and valve morphogenesis and development (Timmerman et al., 2004). Disruption of key Notch pathway components, including receptors (e.g., *Notch1*), ligands (e.g., *Jagged1*), transcriptional repressor mediators (e.g., *Hey2, Hes1*) and mediators of receptor activation (e.g., *Adam10*), results in the complex structural defects commonly observed in TOF in both humans and mice (Garg et al., 2005; Kerstjens-Frederikse et al., 2016). Genetically engineered mouse models of the various Notch pathway mutants recapitulate some of the key features of TOF, including ventricular septal defects (VSD), overriding aorta (OA) and pulmonary stenosis (Kelly, 2024).

##### 3.4.1.1 Hey2^−/−^

The *Hey2* ^*−/−*^ mutant mice develop lethal cardiac malformations and show a spectrum of CHD malformations reminiscent of human conotruncal heart defects and TOF. Notably, all *Hey2* ^−/−^ embryos examined exhibit membranous VSD with OA, yet pulmonary stenosis and OFT obstruction are co-expressed in only approximately 40% of cases (Donovan et al., 2002). While this combination of defects constitutes the core anatomy of TOF, the penetrance of the defects is background strain dependent, with mixed backgrounds showing lower incidence of full TOF (Sakata et al., 2006). In severe cases, *Hey2* ^*−/−*^ embryos present with tricuspid valve atresia, mitral valve thickening, and septation defects not usually found in TOF, such as atrial septal defects (ASD) (Donovan et al., 2002; Sakata et al., 2006). Right and left ventricular hypertrophy is also observed postnatally in *Hey2* ^*−/−*^ mutant mice, though it is seldom documented due to the early mortality of this strain. Overall, defects in *Hey2* ^*−/−*^ mutant mice resemble those caused by *JAGGED1* gene mutations in humans (Alagille syndrome) (McElhinney et al., 2002), typically displaying three of four TOF features (VSD, OA and OFT obstruction), with rare cases meeting all four criteria. Although *HEY2* variants have been identified in human CHD patients, the evidence for *HEY2* as a direct cause of human TOF remains limited, highlighting a discordance between mouse and human phenotypes that is a recognised challenge in modelling conotruncal defects. *HEY2* proteins are transcriptional repressors downstream of Notch signalling that bind to the cardiac transcription factor *GATA4*, resulting in the inhibition of *GATA4*-dependent gene activation (Xiang et al., 2006).

##### 3.4.1.2 Notch1^+/−^;Nos3^−/−^

Compound mutants heterozygous for *Notch1* and null for *Nos3* are another mouse model related to *Notch* family signalling that partially recapitulates the core features of TOF. Individually, these mutations are not suitable to study TOF, as *Notch1* ^*−/−*^embryos die at E10 due to severe vascular defects before OFT septation occurs (Conlon et al., 1995), and *Notch1* ^*+/−*^ mice exhibit subtle to no cardiac developmental defects (Matos-Nieves et al., 2021). In contrast, although *Nos3* ^*−/−*^ mice exhibit VSD and semilunar valve thickening, the frequency of reported OA, pulmonary stenosis, and right ventricular hypertrophy is not consistent (Liu and Feng, 2012). In combination, these mutations synergise to cause a spectrum of OFT malformations including bicuspid aortic valve (BAV) and semilunar valve stenosis (affecting the aortic and/or pulmonary valves), membranous VSD and OA at E18.5 (Matos-Nieves et al., 2021; Koenig et al., 2016). *Notch1* ^*+/−*^ *;Nos3* ^−/−^ mice also exhibit significant embryonic and perinatal lethality (∼65%) by postnatal day 10, limiting the progression to observable right ventricular hypertrophy that would likely occur as a secondary consequence of sustained OFT obstruction (Bosse et al., 2013). Importantly, not only does the cardiovascular phenotype exhibited by the *Notch1* ^*+/−*^ *;Nos3* ^*−/−*^ mice satisfy three out of the four classic components of TOF (VSD, OA, and thickened pulmonary valve leaflets consistent with, but not demonstrated as, valvular pulmonary stenosis), but it also aligns with the defects observed in human patients harbouring *Notch1* mutations (Garg et al., 2005; Kerstjens-Frederikse et al., 2016). This dual-mutant mouse model underscores the important role of *Notch1* signalling in the endocardium during OFT development, and its contribution to TOF-associated cardiac malformations when disrupted in combination with *Nos3*.

##### 3.4.1.3 *Jagged1* (*J1^ECKO^*)

During heart development, the Notch ligand Jagged1 (*Jag1*) is expressed in the coronary arteries, second heart field and neural crest cells (de la Pompa and Epstein, 2012). Beyond its established role in Alagille syndrome, *JAG1* point variants have also been reported in non-syndromic TOF, as reviewed elsewhere (Althali and Hentges, 2022). Briefly, functionally relevant *JAG1* sequence changes were found in around 3% (2/94) of non-syndromic TOF cases in one cohort, and the c.765C>T variant was strongly associated with TOF in an Iranian population (60.2% versus 5.7% in controls) (Althali and Hentges, 2022). The mechanism linking *JAG1* alterations to TOF remains unclear, which makes the gene an informative target for study in the mouse. Earlier compartment-specific inactivation of *Jag1* in mice, caused lethality by E10.5 (High et al., 2009), so to examine *Jag1* at later stages, the gene was deleted in the endothelium using a *VE-Cadherin-Cre* driver (J1^ECKO^) (Hofmann et al., 2012). This mosaic deletion reproduces the cardiac component of Alagille syndrome, with J1^ECKO^ mutant embryos developing several hallmarks of TOF, namely, VSD, OA and RVH, alongside valve malformations with stenosis and coronary vessel anomalies (Hofmann et al., 2012). Of 46 embryos and neonates examined, 47.8% had at least one cardiac defect, with overriding aorta being the most common (30.4%) (Hofmann et al., 2012). Penetrance is variable, reflecting mosaic activity of the *VE-Cadherin-Cre* driver, and many mutants survive to adulthood, where half develop valve calcification that lies outside the developmental tetrad (Hofmann et al., 2012). This model remains highly informative, as the principal Alagille gene is tied directly to TOF-like anatomy, and RVH, which is rarely captured in embryonic-lethal models, is observed in surviving animals.

#### 3.4.2 VEGF^120/120^

The VEGF^120/120^ mouse model, which was engineered to express only the VEGF120 (Vascular Endothelial Growth Factor-A) protein isoform, is an indirect Notch pathway model that partially recapitulates TOF through dysregulated *Vegf-Notch* signalling interactions. Initially, this model was created to investigate the role of *VEGF* in skeletal development (Zelzer et al., 2002), however it was later discovered to have high susceptibility for development of TOF-like defects including pulmonary stenosis and VSD (van den Akker et al., 2007). More specifically, VEGF^120/120^ mouse embryos develop malalignment of the outlet septum resulting in subaortic VSD with dextroposition of the aorta. van den Akker (2007) classified this as double outlet right ventricle in 32% of embryos (10/31) at E14.5–E19.5, and, where right ventricular outflow tract stenosis was also present, as TOF in 29% (9/31) (van den Akker et al., 2007). Pulmonary trunk and artery hypoplasia was frequent (61%, 19/31), and in severe cases progressed to near-complete atresia of the pulmonary trunk (van den Akker et al., 2007). Additionally, this model is particularly relevant for studying severe TOF with pulmonary atresia (TOF/PA) in humans, as a subset of VEGF^120/120^ mouse embryos progress from having pulmonary stenosis to pulmonary atresia as they mature *in utero* (Rammeloo et al., 2015). This combination of VSD, OA, and severe pulmonary stenosis is directly linked to aberrant VEGF signalling and subsequent ectopic activation of *Notch1* and *Jagged2* in the OFT, which causes malalignment of the outlet septum and overgrowth of the endocardial cushions (van den Akker et al., 2007). In summary, the VEGF^120/120^ mouse model reproduces three of the four core TOF features: VSD, OA and pulmonary stenosis, and uniquely captures progression to pulmonary atresia in a subset of embryos, though right ventricular hypertrophy remains unobserved due to perinatal lethality.

#### 3.4.3 Robo1^I270T^

*ROBO* signalling receptors, through their interaction with *SLIT* ligands, provide essential guidance cues for cNCC migration into the outflow tract, facilitating proper ventricular septation and the formation of atrioventricular and semilunar valves (Medioni et al., 2010). At a mechanistic level, *Slit-Robo* signalling also modulates NOTCH activity during cushion formation, demonstrated by reduced expression of *Notch* receptors and downstream targets *Hey/Hes* in endocardial cushions of *Robo1* ^*−/−*^ mice (Mommersteeg et al., 2015). The *Robo1* ^*I270T*^ mouse model, identified through an ENU mutagenesis screen, was reported to exhibit membranous VSD, OA, and DORV, partially recapitulating the OFT malalignment and septal defects characteristic of TOF (Kruszka et al., 2017). Interestingly, newborn mutant *Robo1* ^*I270T*^ mice also presented with ASD and AVSD, accompanied by craniofacial anomalies including cleft palate and micrognathia, mirroring the cardiac and craniofacial features reported in human patients with *de novo ROBO1* variants (Kruszka et al., 2017). To date, only one publication has described the phenotype of the *Robo1* ^*I270T*^ mouse model, and due to absence of data on survival and lethality, as well as pulmonary stenosis and right ventricular hypertrophy, definite conclusions about these aspects cannot be drawn. Despite these limitations, the phenotypic overlap between the *Robo1* ^*I270T*^ mouse model and human *ROBO1* variant carriers supports *ROBO1* as a candidate gene in conotruncal CHD, though further studies are needed to firmly establish its causative role in TOF.

#### 3.4.4 Cited2^−/−^

*CITED2* is a transcriptional modulator essential for proper heart development, including cardiac morphogenesis and blood vessel formation (Yin et al., 2002; Bamforth et al., 2001). The *Cited2* ^*−/−*^ mouse is an established genetic model that exhibits a spectrum of conotruncal defects, including OFT malformations characteristic of TOF. *Cited2* ^*−/−*^ embryos display severe malformations including exencephaly and cardiac defects such as ASD, VSD, OA and OFT malalignment including DORV or truncus arteriosus (TA) (Bamforth et al., 2001). The timing of embryonic lethality in this model is variable and dependent on the severity of the cardiac defects. While many *Cited2* ^*−/−*^ embryos die in mid-gestation (E10.5-E14.5) due to severe cardiac malformations, a subset with less severe phenotype survive to later embryonic stages (Bamforth et al., 2001). Importantly, ventricular hypertrophy has been reported in embryos that survive to late gestation (E17.5) (Bamforth et al., 2001). However, the original study does not explicitly state whether this hypertrophy was observed concurrently with the three other key features of TOF (VSD, OA, and OFT obstruction) in the same embryos (Yin et al., 2002; Bamforth et al., 2001). Thus, while the *Cited2* ^*−/−*^ model recapitulates individual hallmark features of TOF, the full tetrad has not been documented as occurring simultaneously in a single embryo. Mutations in the coding and promoter regions of *CITED2* have been identified in human patients with TOF, mirroring the molecular defects seen in *Cited2* ^*−/−*^ mice including OFT and septal malformations (Li et al., 2012; Chen et al., 2022). Loss of *CITED2* function in both humans and mice disrupts left-right patterning via *Nodal/PITX2C*, hypoxia response via *HIF-1α/VEGF*, and *TFAP2*-mediated transcription, resulting in OFT malalignment, septal defects and laterality anomalies (Xu et al., 2014). While *Cited2* null mice display highly penetrant TOF-like defects, mutant mice carrying the T166N variant in the CITED2 serine-glycine-rich domain identified from human CHD patients show no obvious heart abnormalities, suggesting that additional genetic or environmental modifiers are needed for the manifestation of TOF (Chen et al., 2012).

#### 3.4.5 Fog2^−/−^

The *Fog2 (Zinc Finger Protein, Multitype 2*) knockout mouse model is another well-characterised example of TOF-like pathology in the mouse. By partnering with its cofactor Gata4, Fog2 drives EndMT in endocardial cells for cushion formation and cNCC-dependent OFT septation (Tevosian et al., 2000). *Fog2* ^*−/−*^ mice present with a constellation of cardiac malformations including extremely thin ventricular myocardium and common atrioventricular canal, whereby the endocardial cushions fail to fuse properly, resulting in contiguous ASD and VSD (Tevosian et al., 2000). Moreover, at E13.5, *Fog2* ^*−/−*^ embryos present with subpulmonic stenosis and misalignment of the OFT with an OA, partially recapitulating the core anatomical features of TOF (Tevosian et al., 2000). The caveat of this model is that because of the cardiac defects, embryos die between E12.5-E14.5 and therefore right ventricular hypertrophy, which is typically seen in postnatal TOF, cannot be observed. Consistent with the phenotype observed in *Fog2* knockout mice, FOG2 variations such as E30G and S657G have been implicated in human TOF, though the limited number of variation-positive patients precludes a detailed genotype-phenotype correlation (Pizzuti et al., 2003). In summary, *Fog2* is essential for normal cardiac septation, and its loss leads to TOF-like conotruncal defects, though early embryonic lethality prevents complete recapitulation of the full TOF tetrad.

#### 3.4.6 Tbx1^neo2^

TBX1 haploinsufficiency, most commonly arising from heterozygous microdeletion of the 22q11.2 chromosomal region, is a well-established cause of conotruncal defects including TOF in humans. Patients with TBX1 haploinsufficiency present with a range of cardiac malformations including aortic arch defects, VSD, TA and TOF (Guo et al., 2011). In mice, homozygous *Tbx1* ^*−/−*^ mutants display severe cardiac defects including persistent TA with VSD, whereas *Tbx1* ^*+/−*^ mice show a milder phenotype typically limited to interrupted aortic arch (IAA) and other aortic arch defects (Vitelli et al., 2002). However, these models do not faithfully reproduce the full variability of defects seen in human patients. To address this dosage discrepancy, the *Tbx1* ^*neo2*^ hypomorphic model was generated, expressing only 20% of normal *Tbx1* mRNA. Indeed, *Tbx1* ^*Neo2/Neo*^ mutant mice exhibit cardiac defects similar to human 22q11.2 syndrome including DORV, IAA, perimembranous VSD, and OFT defects such as TA and TOF (Zhang and Baldini, 2008; Majumdar et al., 2021). Notably, mice require an 80% reduction in *Tbx1* expression to manifest TOF-like malformations, whereas human patients appear considerably more sensitive to *TBX1* loss, with a 50% reduction due to 22q11.2 deletion sufficient to cause TOF with variable penetrance (Zhang and Baldini, 2008). Mechanistically, loss of Tbx1 disrupts multiple pathways critical for OFT development, including *Gbx2-Slit* signalling which affects cNCC migration in the pharyngeal arches (Calmont et al., 2009), FGF-mediated progenitor proliferation, and Wnt5a/PCP-directed cell deployment (Sinha et al., 2015). This results in inadequate OFT elongation, malformed cardiac cushions, and great artery misalignment, a series of developmental perturbations that explains phenotypes like TOF, DORV, and truncus arteriosus. In summary, the *Tbx1* ^*Neo2/Neo*^ mouse model reproduces TOF-like anatomy and highlights the finely tuned gene-dosage sensitivity of *TBX1*, though the requirement for extreme *Tbx1* reduction in mice compared to humans underscores the species differences that remain a challenge in modelling human conotruncal defects.

#### 3.4.7 *Shh* ^−/−^

Sonic hedgehog (*Shh*) has a broad patterning role that extends to the heart, where it is required for normal conotruncal development. *Shh* ^−/−^ embryos develop a severe conotruncal phenotype resembling TOF with pulmonary atresia. All late-gestation mutants examined (E16.5–E18.5) showed abnormal cardiovascular development, with VSD, OA, and thickened myocardium, suggesting hypertrophy (Smoak et al., 2005). Additional anomalies, including a common atrium, right aortic arch, shortened atrioventricular valves and anomalous pulmonary venous return, lie outside the tetrad and reflect a broader disruption of cardiac development (Smoak et al., 2005). Perinatal lethality, together with severe craniofacial and pharyngeal defects, prevents assessment of postnatal right ventricular hypertrophy (Smoak et al., 2005). Mechanistically, SHH signalling is required within the pharyngeal endoderm to support SHF proliferation and cNCC survival. *Shh* loss leads to depletion of arterial pole progenitors and, in more severe contexts, impaired NCC contribution to the outflow tract, compromising OFT elongation and septation (Goddeeris et al., 2007; Dyer and Kirby, 2009). Notably, pulmonary atresia occurred even in the presence of a septum, suggesting that SHF progenitor deficiency alone is sufficient to drive this aspect of the phenotype. Detailed mechanistic insights have been extensively reviewed elsewhere (Dyer and Kirby, 2009). Although *Shh* ^−/−^ mice develop a conotruncal phenotype closely resembling TOF with pulmonary atresia, SHH mutations in humans are most commonly associated with non-syndromic holoprosencephaly rather than isolated TOF. Hedgehog pathway disruption may contribute to CHD more broadly (Li et al., 2015), but the role of direct SHH mutation in isolated TOF remains poorly defined.

#### 3.4.8 *Mef2c* ^AHF−KO^

The transcription factor MEF2C acts in the anterior second heart field (AHF) and is necessary for normal cardiac morphogenesis including rightward looping and OFT alignment, with mice lacking *Mef2c* exhibiting early embryonic lethality (Lin et al., 1997). Conditional deletion of *Mef2c* in the AHF in mice (*Mef2c* ^AHF−KO^), produced a spectrum of alignment defects, with around half of mutants dying shortly after birth from cyanosis (Barnes et al., 2016). Of 36 mutants examined, five displayed a TOF-like phenotype, with overriding aorta, a stenotic or atretic pulmonary artery, membranous VSD and a hypertrophic right ventricle (Barnes et al., 2016). Because *Mef2c* ^AHF−KO^ mice are not embryonic lethal, this is one of the few models in which right ventricular hypertrophy is documented as part of a TOF-like phenotype rather than inferred. The remaining mutants showed isolated valve defects, isolated VSD, VSD with DORV, or VSD with dextro-transposition of the great arteries (d-TGA). MEF2C directly activates *Tdgf1*, which encodes the Nodal co-receptor Cripto, through an AHF-restricted enhancer, and *Tdgf1* expression in the OFT is almost abolished in its absence (Barnes et al., 2016). In humans, MEF2C-related disorders are rare, and are more commonly characterized by intellectual disability, developmental delay, and impaired motor development (Qiao et al., 2017; Cooley Coleman et al., 2021). Congenital heart disease, including TOF, has been reported but is not the dominant clinical feature (Qiao et al., 2017; Cooley Coleman et al., 2021).

#### 3.4.9 Wnt signalling pathway

The cardiac malformations of TOF, especially conotruncal defects, are generally thought to arise from morphogenetic disturbances in the SHF and cardiac neural crest cells (NCCs) (Waldo et al., 2005). Previous studies of the molecular mechanisms of conotruncal heart defects demonstrated that the Wnt-signalling pathway affects heart development by regulating the proliferation, differentiation and migration of cardiac progenitor cells, especially in regulating the development of the cardiac outflow tract (OFT) (Ai et al., 2007; Klaus et al., 2012). Multiple mouse models with genetic perturbations in components of both canonical and non-canonical Wnt signalling pathway recapitulate conotruncal malformations resembling TOF, including overriding or mispositioned great arteries, VSD, and OFT abnormalities.

##### 3.4.9.1 Lrp6 ^−/−^

As an important Wnt co-receptor, Lrp6 is essential for canonical Wnt/β-catenin signalling. Most of *Lrp6* ^−/−^ mice survive until birth but display significant heart defects. Homozygous embryos exhibit severe conotruncal abnormalities from E11.5, progressing to VSD, TGA and DORV at E14.5 and E16.5 (Song et al., 2010; Bryja et al., 2009), consistent with a severe conotruncal malalignment phenotype characterised by substantial aortic override rather than classical TOF. These *Lrp6* KO mutants also show loss of cardiac neural crest cells (cNCCs) in the pharyngeal arches (Song et al., 2010). Although pulmonary artery stenosis was not reported, the important role of cNCCs in the development of the OFT and the correlation between OFT cushion defects and pulmonary artery defects (Yelbu et al., 2002), suggests that failure in OFT septation contributes to the developmental origin of conotruncal defects relevant to TOF.

##### 3.4.9.2 Ctnnb1

Due to the essential role of β-catenin in early embryogenesis, global knockout or severe knockdown *Ctnnb1* models are embryonic lethal prior to gastrulation (Rudloff and Kemler, 2012). Deficiency of β-catenin in mesoderm-derived pharyngeal mesenchyme, as modelled in *Ctnnb1* ^flox/flox^; Dermo1-Cre mice, leads to severe conotruncal malformations, characterized by hypoplastic pulmonary artery branches, abnormal aortic arch, atrial and ventricular septal defects (100% penetrance), double outlet right ventricle (25% penetrance), and overriding aorta (50% penetrance) by E16.5 (Huh and Ornitz, 2010), closely resembling the structural features of TOF, although right ventricular hypertrophy was not reported. In comparison, conditional deletion of β-catenin in second SHF progenitors using *Ctnnb1* ^flox/flox^ *;* Mef2c-Cre mice revealed a more restricted phenotype, with reduced right ventricular (RV) size and shortened OFT, accompanied by failed septation of the pulmonary trunk and aorta in the distal OFT at E12.5 (Ai et al., 2007). This latter model underscored that Wnt/β-catenin signalling promotes SHF expansion and suppresses FHF development (Ai et al., 2007).

Collectively, Wnt/β-catenin signalling within SHF progenitors primarily contributes to the development of the RV and OFT, being particularly essential for distal OFT septation, but appears not to be required for atrial and ventricular septation. In contrast, loss of β-catenin in mesoderm-derived pharyngeal mesenchyme results in more extensive defects. These findings are consistent with the contributions of these cell populations during normal cardiac development and highlight the spatially distinct roles of canonical Wnt signalling during cardiogenesis. Considering that VSD is the critical lesion producing the mixing of oxygenated and deoxygenated blood in TOF, the *Ctnnb1* ^flox/flox^; Dermo1-Cre model provides a more suitable system for early embryonic analysis of TOF.

##### 3.4.9.3 Wnt5a

As a critical component of the non-canonical Wnt signalling pathway, tightly regulated expression of Wnt5a is indispensable for normal outflow tract (OFT) morphogenesis. Global deletion of *Wnt5a* in mice results in perinatal lethality accompanied by severe cardiac malformations (Yamaguchi et al., 1999), most notably conotruncal anomalies (Schleiffarth et al., 2007). Nearly all *Wnt5a*-null embryos examined between E13.5 and E17.5 display VSD in conjunction with persistent truncus arteriosus (PTA), while approximately half of the mutants also exhibit aortic arch abnormalities, including interruption of the aortic arch (IAA) and right-sided aortic arch (AoR) (Sinha et al., 2015; Schleiffarth et al., 2007). Only a minority of embryos present with transposition of the great arteries (TGA) or double outlet right ventricle (DORV) (Schleiffarth et al., 2007). The high penetrance of PTA and VSD strongly suggests that *Wnt5a* deficiency perturbs the entire program of OFT development, disrupting both proximal septation of the outlet septum and its subsequent contribution to the ventricular septum (Sinha et al., 2015; Schleiffarth et al., 2007). However, by E9.5, a shortened OFT phenotype was also observed in mice with *Wnt5a* overexpression in SHF progenitors (Li et al., 2016).

WNT5A regulates SHF cell polarity through the PCP pathway, such that in *Wnt5a*-deficient embryos the caudal splanchnic mesoderm (SpM) fails to contribute SHF cells rostrally, whereas *Wnt5a* overexpression causes SHF cells to accumulate in a bulge within the SpM, thereby recapitulating OFT shortening and conotruncal defects (Sinha et al., 2015; Li et al., 2016; Li et al., 2019). Subsequent rescue of the *Wnt5a*-deficient phenotype by the PCP effector *Daam1* further substantiates this mechanism. In addition to its role in the SHF, *Wnt5a* expression in the pharyngeal mesoderm also indicates a link to cardiac neural crest cells (NCCs). While WNT5A does not markedly affect NCC migration, it may regulate cardiac NCC condensation into the aortopulmonary septum through Wnt/Ca^2+^ signalling (Schleiffarth et al., 2007). Overall, the phenotypes observed in *Wnt5a* ^−/−^ embryos overlap with the conotruncal anomalies commonly seen in TOF, particularly VSD and OFT septation defects. Given that TBX1 acts as an upstream transcriptional activator of *Wnt5a*, the comparable defects observed in *Tbx1* mutant embryos further underscore the critical role of *Wnt5a* in orchestrating conotruncal morphogenesis (Sinha et al., 2015).

##### 3.4.9.4 Wnt11

As another non-canonical Wnt ligand, WNT11 shares overlapping yet distinct functions with WNT5A (Cohen et al., 2012), also acting through both the PCP and Wnt/Ca^2+^ pathways to coordinate cardiogenesis and conotruncal morphogenesis. *Wnt11* knockout mice display highly penetrant embryonic lethality, particularly on the 129S genetic background (Nagy et al., 2010). Although cardiac looping proceeds normally, the earliest cardiac defect appears at E9.5 as a shortened OFT (Zhou et al., 2007). By E10.5, mutant embryos exhibit dilated ventricular chambers, thinning of the ventricular wall, and hypoplastic, irregularly arranged trabeculae (Nagy et al., 2010). These embryos develop fully penetrant defects in both the OFT and the dorsal mesenchymal protrusion (DMP), with abnormal OFT rotation leading to DORV and transposition of the great arteries (TGA) accompanied by VSD at E14.5 (van Vliet et al., 2017).

Lineage-specific deletion further demonstrates that ablation of *Wnt11* in both cTnT-Cre-marked myocardial cells and Isl1-Cre-marked SHF progenitors recapitulates the cardiac defects observed in germline null embryos, whereas deletion in neural crest, endocardial, or epicardial lineages does not produce detectable phenotypes (van Vliet et al., 2017). These findings indicate that *Wnt11*-null mice model severe OFT malalignment driven by SHF-derived myocardial populations, representing a non–neural crest mechanism of conotruncal defect formation.

##### 3.4.9.5 Vangl2

*Vangl2* is a key component of the non-canonical Wnt signalling pathway. *Looptail* (*Lp*) mutants carrying *Vangl2* loss-of-function alleles exhibit similar conotruncal malformations, including DORV and VSD at E13.*5* (Henderson et al., 2001). Some *Vangl2* ^*Lp/Lp*^ mice also have abnormal heart looping and aortic arch abnormalities, reminiscent of the frequent right aortic arch in TOF patients (Henderson et al., 2001). At E14.5, *Vangl2* ^*flox/flox*^, Sox2-Cre embryos fully recapitulate the *Vangl2* ^*Lp/Lp*^ phenotype, including severe craniorachischisis, DORV, VSD and pharyngeal arch remodelling defects, which are indistinguishable from *Vangl2* ^*Lp/Lp*^ embryos, confirming that VANG2 loss is responsible for these malformations (Ramsbottom et al., 2014). In contrast, *Vangl2* ^flox/flox^ *;* Wnt1-Cre did not result in any heart or OFT abnormalities; these embryos appear phenotypically normal at E14.5 and postnatally (Ramsbottom et al., 2014). These results suggest that the OFT defects observed in *Lp* mutants might originate from the neuroepithelium instead of the cNCC lineage, indicating that in cNCCs, *Vangl2* or the non-canonical PCP pathway may not be essential for proper OFT formation.

In summary, both canonical and non-canonical Wnt signalling pathways are indispensable for SHF, cNCCs and mesenchyme contributions to OFT septation and ventricular septum formation. Across these models, disruption of Wnt signalling consistently results in severe conotruncal malformations, frequently recapitulating key structural features associated with TOF. Importantly, lineage-specific models indicate that these defects arise through distinct mechanisms, providing complementary frameworks for dissecting TOF pathogenesis.

### 3.5 Cilia-associated oligogenic mouse model

Recently, an oligogenic mouse study has extended the traditional monogenic framework of conotruncal heart defects by demonstrating that combined disruption of multiple cilia-associated genes can synergistically impair OFT development (Zhou et al., 2025). Six individual mutant mouse lines targeting cilia-associated candidate genes identified from TOF patient sequencing data were generated, in which homozygous animals exhibited perinatal lethality accompanied by a spectrum of OFT and septal defects, including overriding aorta with VSD and ASD. *Pkhd1l1* ^−/−^ homozygous mice showed the highest frequency of these defects. In a subset of embryos, concomitant pulmonary artery narrowing further recapitulated TOF-like features.

In contrast, heterozygous mice carrying single ciliary mutations exhibited milder and incompletely penetrant cardiac abnormalities, consistent with a limited effect of individual ciliary gene disruption on OFT morphogenesis (Zhou et al., 2025). However, pairwise crosses generating double-heterozygous mice resulted in a marked increase in both the incidence and severity of conotruncal anomalies, particularly overriding aorta (Zhou et al., 2025).

Transcriptomic analysis of mouse embryonic hearts further identified consistent downregulation of Hedgehog signalling across all ciliary gene mutant models, accompanied by reduced expression of the downstream cardiac transcription factors *Gata4* and *Nkx2-5* (Zhou et al., 2025).

Together, these findings highlight ciliary function as a critical contributor to conotruncal defect pathogenesis and, importantly, demonstrate that combinatorial disruption of multiple ciliary genes provides a more powerful and biologically relevant modelling strategy than single-gene mutations alone. This oligogenic framework further suggests that strategic crosses between previously described monogenic models may yield improved representations of the genetic complexity and phenotypic variability observed in human TOF.

### 3.6 Summary of mouse models

Taken together, the monogenic and oligogenic mouse models discussed above show that distinct genetic perturbations can converge on a limited set of morphogenetic processes required for OFT alignment and septation. In most cases, individual models reproduce discrete components of classical TOF rather than the complete tetrad. It is also important to recognise that, in contrast to clinical CHD where structured classification systems are routinely applied (e.g., IPCCC, now incorporated into ICD-11), there is currently no universally adopted framework for phenotyping and reporting mouse CHDs (Henderson et al., 2024). As a result, comparable anatomical lesions may be described or categorised differently across laboratories, limiting direct comparison between mouse models and human diagnoses. Standardisation will become increasingly relevant as more complex genetic models are used to capture oligogenic contributions in human TOF.

An additional challenge highlighted by several murine models is the apparent difference in gene dosage sensitivity between experimental systems and human disease. This concept is exemplified by the *Tbx1* models (Vitelli et al., 2002; Zhang and Baldini, 2008), the *Notch1* ^*+/−*^ *;Nos3* ^*−/−*^ mutant (Matos-Nieves et al., 2021), and the cilia-associated oligogenic models (Zhou et al., 2025) discussed above, all of which require a stronger genetic insult in mice than is typically observed in human TOF to generate comparable cardiac phenotypes.

Several factors may contribute to this interspecies difference. Loss-of-function phenotypes in mice are often buffered by genetic redundancy and by compensatory upregulation of related genes, which can mask the effect of disrupting a single gene (Barbaric et al., 2007). In some cases, a paralogue present in the mouse but absent in humans accounts directly for a milder murine phenotype (Barbaric et al., 2007). The use of inbred strains may add to this discrepancy by removing the sensitising background variation present in human populations, so that a mutation on a uniform genetic background must be more severe to cross the same developmental threshold. This finding would be consistent with the oligogenic models discussed above, in which apparent single-gene haploinsufficiency in humans may in practice reflect a heterozygous variant acting together with additional genetic modifiers (Zhou et al., 2025). However, the stronger genetic perturbations required to generate cardiac phenotypes in mice may also shift the developmental outcome towards more severe conotruncal defects, including persistent truncus arteriosus and severe DORV, rather than TOF itself. This may partly explain why severe OFT malformations are most represented among current murine models of conotruncal defect. A further consideration is that laboratory animals are maintained under controlled conditions. They are not routinely exposed to the environmental insults (Calcaterra et al., 2026), such as gestational hypoxia (Bojórquez Mar et al., 2024), that are thought to contribute to human TOF. The absence of such gene–environment interactions may be one reason why current models do not fully reproduce the human phenotype.

Given that human TOF is genetically heterogeneous and frequently exhibits incomplete penetrance, these findings may suggest that the genetic architecture underlying some TOF cases is not fully captured by traditional single mutation models. Collectively, these observations highlight the importance of considering oligogenic interactions when modelling TOF and suggest that future experimental approaches may need to move beyond traditional monogenic paradigms to better capture the complexity of human disease.

## 4 Future perspectives

Recent technological advances are beginning to complement the experimental models discussed throughout this review. Large-scale patient sequencing continues to expand the number of candidate genes associated with TOF (Zhou et al., 2025; Page et al., 2019), while CRISPR-based screening provides an efficient strategy for prioritising variants for functional investigation. Similarly, multi-omics approaches have improved our understanding of the developmental timing, cellular populations and signalling pathways disrupted during OFT morphogenesis (Tang et al., 2022; Ding et al., 2025; Zulibiya et al., 2023). Integration of patient sequencing data with public single-cell and spatial transcriptomic datasets revealed that most of TOF-associated genes are expressed in myogenic progenitors of the cardiac OFT, particularly conoventricular progenitor populations (Tang et al., 2022). Single-cell and single-nucleus transcriptomic analyses further revealed altered cellular composition and transcriptional reprogramming in TOF hearts (Ding et al., 2025; Zulibiya et al., 2023), including endothelial populations with enhanced EMT potential and activated adhesion/ECM, TGF-β, Wnt and Hippo/YAP signalling (Zulibiya et al., 2023). More recently, integrative multi-omics analyses combining bulk and single-nucleus transcriptomic datasets suggested dysregulated proteostasis, RNA processing and mitochondrial metabolism in RVOT remodelling, (Wang et al., 2026), providing additional molecular hypotheses for future functional investigation. Patient-derived iPSC models further provide a genetically human system in which non-coding variants, copy number variation and complex genetic backgrounds can be investigated in ways that are difficult to achieve using conventional animal models alone. For instance, iPSC-derived cardiomyocytes from patients with TOF reveal transcriptional alterations during cardiomyocyte differentiation (Gr et al., 2020).

Although these emerging technologies provide important insights into the molecular mechanisms underlying TOF, they cannot replace *in vivo* developmental models. Current *in vitro* systems cannot fully reproduce the complex spatiotemporal interactions, haemodynamic forces and tissue morphogenesis that drive normal cardiac development and therefore cannot fully capture the developmental processes leading to TOF. Instead, these approaches are likely to be most valuable when integrated with animal models. Rather than selecting variants for modelling solely based on patient sequencing, future studies may adopt a stepwise strategy to prioritise candidate variants. First, large-scale sequencing can identify candidate genes associated with TOF. Developmental single-cell and spatial transcriptomic datasets can then be used to prioritise genes expressed in OFT-relevant cell populations during the critical stages of cardiac development. Integration with patient-derived multi-omics datasets and functional validation may subsequently identify variants that reproduce the molecular changes observed in affected hearts, providing stronger candidates for animal modelling. Finally, incorporating gene dosage, oligogenic inheritance and regulatory variation into these models may facilitate the development of experimental systems that more closely reflect the molecular and developmental complexity of human TOF.

## 5 Conclusion

Tetralogy of Fallot is fundamentally an outflow tract malalignment disorder. Different models capture complementary pieces of this process. Chick surgery and targeted ablations demonstrate how second heart field and cardiac neural crest contributions elongate and divide the OFT, directly linking impaired addition of distal myocardium to VSD, aortic override, and pulmonary stenosis (Aranega et al., 1985; Wa et al., 2005). Despite Zebrafish and *Xenopus* having simpler chamber anatomy, these models can be used to efficiently test gene function and visualize OFT patterning, and their perturbations can still mirror human TOF pathogenesis (Lee and Saint-Jeannet, 2011).

Within this landscape, mouse genetics remains central to the study of TOF because the four-chamber anatomy and ability to generate precise mutant alleles in the mouse, allows TOF like defects to emerge. Across the mechanisms and pathways emphasized here, including Notch/Jagged, VEGF, TBX1 dosage, canonical and non-canonical Wnt signalling, SLIT–ROBO*,* and ciliary function, important contributions have been made to our understanding of TOF. However, many mouse models display frequent embryonic or perinatal lethality, background dependent penetrance, a tendency toward severe phenotypes (PTA/DORV) or differences in gene level sensitivity that complicate the one-to-one mapping of classic TOF. This may reflect fundamental differences in how murine and human cardiac development responds to genetic perturbation, whereby stronger genetic changes are often required to generate TOF-like phenotypes in mice but may also result in more severe conotruncal defects. Equivalent genetic changes do not necessarily produce equivalent developmental outcomes across species. Instead, the developmental consequences of a given mutation are likely influenced by gene dosage, genetic background and the sensitivity of the underlying developmental programme.

Overall, firstly there is no single model that consistently and reproducibly recapitulates the full constellation of the four features of human TOF within a stable genetic background, although different experimental systems continue to provide complementary insights into disease pathogenesis. Second, future studies should also place greater emphasis on gene dosage, oligogenic inheritance and regulatory variation, which are increasingly recognised as important contributors to the genetic architecture of TOF. Finally, continued integration of animal model research with human data remains essential. In particular, the interacting effects of multiple genetic variants, together with regulatory perturbations arising from non-coding regions, should be incorporated into mechanistic models of TOF. Together, cross-species approaches, supported by emerging technologies such as large-scale human genetics, functional genomics and multi-omics, may provide an opportunity to advance our understanding of conotruncal development. Refining animal models to capture the more subtle developmental perturbations characteristic of human TOF will be critical for reconciling the molecular mechanisms with human genetic variation.

## References

[B1] Ai D. Fu X. Wang J. Lu M.-F. Chen L. Baldini A. (2007). Canonical Wnt signaling functions in second heart field to promote right ventricular growth. Proc. Natl. Acad. Sci. 104 (22), 9319–9324. 10.1073/pnas.0701212104 17519332 PMC1890492

[B2] Alipour S. R. S. van Genuchten W. J. Zandbergen L. M. Henry S. Taverne Y. J. Merkus D. (2023). The right ventricle in tetralogy of fallot: adaptation to sequential loading. Front. Pediatr. 11, 1098248. 10.3389/fped.2023.1098248 37009270 PMC10061113

[B3] Althali N. J. Hentges K. E. (2022). Genetic insights into non-syndromic tetralogy of fallot. Front. Physiology 13, 1012665. 10.3389/fphys.2022.1012665 36277185 PMC9582763

[B4] Aranega A. Egea J. Alvarez L. Arteaga M. (1985). Tetralogy of fallot produced in chick embryos by mechanical interference with cardiogenesis. Anatomical Rec. 213 (4), 560–565. 10.1002/ar.1092130412 4083536

[B5] Bailliard F. Anderson R. H. (2009). Tetralogy of fallot. Orphanet Journal Rare Diseases 4 (1), 2. 10.1186/1750-1172-4-2 19144126 PMC2651859

[B6] Bajpai R. Chen D. A. Rada-Iglesias A. Zhang J. Xiong Y. Helms J. (2010). CHD7 cooperates with PBAF to control multipotent neural crest formation. Nature 463 (7283), 958–962. 10.1038/nature08733 20130577 PMC2890258

[B7] Bamforth S. D. Bragança J. Eloranta J. J. Murdoch J. N. Marques F. I. Kranc K. R. (2001). Cardiac malformations, adrenal agenesis, neural crest defects and exencephaly in mice lacking Cited2, a new Tfap2 co-activator. Nat. Genetics 29 (4), 469–474. 10.1038/ng768 11694877

[B8] Barbaric I. Miller G. Dear T. N. (2007). Appearances can be deceiving: phenotypes of knockout mice. Briefings Funct. Genomics Proteomics 6 (2), 91–103. 10.1093/bfgp/elm008 17584761

[B9] Barnes R. M. Harris I. S. Jaehnig E. J. Sauls K. Sinha T. Rojas A. (2016). MEF2C regulates outflow tract alignment and transcriptional control of Tdgf1. Development 143 (5), 774–779. 10.1242/dev.126383 26811383 PMC4813332

[B10] Bartlett H. L. Sutherland L. Kolker S. J. Welp C. Tajchman U. Desmarais V. (2007). Transient early embryonic expression of Nkx2‐5 mutations linked to congenital heart defects in human causes heart defects in *Xenopus laevis* . Dev. Dynamics An Official Publication Am. Assoc. Anatomists 236 (9), 2475–2484. 10.1002/dvdy.21244 17685485 PMC2078326

[B11] Bertranou E. G. Blackstone E. H. Hazelrig J. B. Turner M. E. Kirklin J. W. (1978). Life expectancy without surgery in tetralogy of fallot. Am. J. Cardiol. 42 (3), 458–466. 10.1016/0002-9149(78)90941-4 685856

[B12] Bojórquez Martínez C. A. García Murillo I. M. Segón Mora S. López Mereles A. (2024). Tetralogy of fallot: hypoxia, the villain of the story? Birth Defects Res. 116 (1), e2279. 10.1002/bdr2.2279 38277413

[B13] Bosse K. Hans C. P. Zhao N. Koenig S. N. Huang N. Guggilam A. (2013). Endothelial nitric oxide signaling regulates Notch1 in aortic valve disease. J. Molecular Cellular Cardiology 60, 27–35. 10.1016/j.yjmcc.2013.04.001 23583836 PMC4058883

[B14] Bryja V. Andersson E. R. Schambony A. Esner M. Bryjová L. Biris K. K. (2009). The extracellular domain of Lrp5/6 inhibits noncanonical Wnt signaling *in vivo* . Mol. Biology Cell 20 (3), 924–936. 10.1091/mbc.e08-07-0711 19056682 PMC2633404

[B15] Calcaterra V. Gervasoni C. Ceriani C. Mannarino S. Lanzotti A. Puricelli F. (2026). Maternal–fetal microRNA axis in congenital heart disease: implications for tetralogy of fallot. Front. Cardiovasc. Med. 13, 1821322. 10.3389/fcvm.2026.1821322 42131094 PMC13161119

[B16] Calmont A. Ivins S. Van Bueren K. L. Papangeli I. Kyriakopoulou V. Andrews W. D. (2009). Tbx1 controls cardiac neural crest cell migration during arch artery development by regulating Gbx2 expression in the pharyngeal ectoderm. Development 136 (18), 3173–3183. 10.1242/dev.028902 19700621 PMC2730371

[B17] Chen C. Bentham J. Cosgrove C. Braganca J. Cuenda A. Bamforth S. D. (2012). Functional significance of SRJ domain mutations in CITED2.PLoS One 7 (10), e46256. 10.1371/journal.pone.0046256 23082118 PMC3474824

[B18] Chen Z. Chen H.-X. Hou H.-T. Yin X.-Y. Yang Q. He G.-W. (2022). Pathophysiological role of variants of the promoter region of CITED2 gene in sporadic tetralogy of fallot patients with cellular function verification. Biomolecules 12 (11), 1644. 10.3390/biom12111644 36358994 PMC9687598

[B19] Cohen E. D. Miller M. F. Wang Z. Moon R. T. Morrisey E. E. (2012). Wnt5a and Wnt11 are essential for second heart field progenitor development. Development 139 (11), 1931–1940. 10.1242/dev.069377 22569553 PMC3347685

[B20] Conlon R. A. Reaume A. G. Rossant J. (1995). Notch1 is required for the coordinate segmentation of somites. Development 121 (5), 1533–1545. 10.1242/dev.121.5.1533 7789282

[B21] Cooley Coleman J. A. Sarasua S. M. Boccuto L. Moore H. W. Skinner S. A. DeLuca J. M. (2021). Comprehensive investigation of the phenotype of MEF2C‐related disorders in human patients: a systematic review. Am. J. Med. Genet. Part A 185 (12), 3884–3894. 10.1002/ajmg.a.62412 34184825

[B22] de la Pompa J. L. Epstein J. A. (2012). Coordinating tissue interactions: notch signaling in cardiac development and disease. Dev. Cell 22 (2), 244–254. 10.1016/j.devcel.2012.01.014 22340493 PMC3285259

[B23] Ding Y. Zhu J. Xu G. Cheng Q. Zhu C. (2025). Single-cell RNA-seq analysis of hearts in patients with fetal tetralogy of fallot. Cardiology 150 (2), 221–231. 10.1159/000540406 39097963

[B24] Donovan J. Kordylewska A. Jan Y. N. Utset M. F. (2002). Tetralogy of fallot and other congenital heart defects in Hey2 mutant mice. Curr. Biology 12 (18), 1605–1610. 10.1016/s0960-9822(02)01149-1 12372254

[B25] Dyer L. A. Kirby M. L. (2009). The role of secondary heart field in cardiac development. Dev. Biology 336 (2), 137–144. 10.1016/j.ydbio.2009.10.009 19835857 PMC2794420

[B26] Ganigara M. Sagiv E. Buddhe S. Bhat A. Chikkabyrappa S. M. (2021). “Tetralogy of fallot with pulmonary atresia: anatomy, physiology, imaging, and perioperative management,” Seminars in Cardiothoracic and Vascular Anesthesia (CA: Los Angeles, CA: SAGE Publications Sage). 10.1177/1089253220920480 32450763

[B27] Garg V. Muth A. N. Ransom J. F. Schluterman M. K. Barnes R. King I. N. (2005). Mutations in NOTCH1 cause aortic valve disease. Nature 437 (7056), 270–274. 10.1038/nature03940 16025100

[B28] Geva T. Wald R. M. Bucholz E. Cnota J. F. McElhinney D. B. Mercer-Rosa L. M. (2024). Long-term management of right ventricular outflow tract dysfunction in repaired tetralogy of fallot: a scientific statement from the American heart association. Circulation 150 (25), e689–e707. 10.1161/cir.0000000000001291 39569497

[B29] Goddeeris M. M. Schwartz R. Klingensmith J. Meyers E. N. (2007). Independent requirements for Hedgehog signaling by both the anterior heart field and neural crest cells for outflow tract development. Development 134 (8), 1593–1604. 10.1242/dev.02824 17344228

[B30] Grunert M. Appelt S. Schönhals S. Mika K. Cui H. Cooper A. (2020). Induced pluripotent stem cells of patients with tetralogy of fallot reveal transcriptional alterations in cardiomyocyte differentiation. Sci. Rep. 10 (1), 10921. 10.1038/s41598-020-67872-z 32616843 PMC7331606

[B31] Guo T. McDonald‐McGinn D. Blonska A. Shanske A. Bassett A. S. Chow E. (2011). Genotype and cardiovascular phenotype correlations with TBX1 in 1,022 velo‐cardio‐facial/digeorge/22q11. 2 deletion syndrome patients. Hum. Mutation 32 (11), 1278–1289. 10.1002/humu.21568 21796729 PMC3196824

[B32] Henderson D. J. Conway S. J. Greene N. D. Gerrelli D. Murdoch J. N. Anderson R. H. (2001). Cardiovascular defects associated with abnormalities in midline development in the Loop-tail mouse mutant. Circulation Research 89 (1), 6–12. 10.1161/hh1301.092497 11440971

[B33] Henderson D. J. Alqahtani A. Chaudhry B. Cook A. Eley L. Houyel L. (2024). Beyond genomic studies of congenital heart defects through systematic modelling and phenotyping. Dis. Models and Mech. 17 (11), dmm050913. 10.1242/dmm.050913 39575509 PMC11603121

[B34] High F. A. Zhang M. Proweller A. Tu L. Parmacek M. S. Pear W. S. (2007). An essential role for Notch in neural crest during cardiovascular development and smooth muscle differentiation. J. Clinical Investigation 117 (2), 353–363. 10.1172/JCI30070 17273555 PMC1783803

[B35] High F. A. Jain R. Stoller J. Z. Antonucci N. B. Lu M. M. Loomes K. M. (2009). Murine Jagged1/Notch signaling in the second heart field orchestrates Fgf8 expression and tissue-tissue interactions during outflow tract development. J. Clinical Investigation 119 (7), 1986–1996. 10.1172/JCI38922 19509466 PMC2701882

[B36] Hofmann J. J. Briot A. Enciso J. Zovein A. C. Ren S. Zhang Z. W. (2012). Endothelial deletion of murine Jag1 leads to valve calcification and congenital heart defects associated with Alagille syndrome. Development 139 (23), 4449–4460. 10.1242/dev.084871 23095891 PMC3509736

[B37] Howe K. Clark M. D. Torroja C. F. Torrance J. Berthelot C. Muffato M. (2013). The zebrafish reference genome sequence and its relationship to the human genome. Nature 496 (7446), 498–503. 10.1038/nature12111 23594743 PMC3703927

[B38] Huh S.-H. Ornitz D. M. (2010). β-catenin deficiency causes DiGeorge syndrome-like phenotypes through regulation of Tbx1. Development 137 (7), 1137–1147. 10.1242/dev.045534 20215350 PMC2835329

[B39] Intarapat S. Stern C. D. (2013). Chick stem cells: current progress and future prospects. Stem Cell Research 11 (3), 1378–1392. 10.1016/j.scr.2013.09.005 24103496 PMC3989061

[B40] Kauling R. M. Unluturk S. Cuypers J. van den Bosch A. E. Hirsch A. Pelosi C. (2025). Long term outcome after surgical tetralogy of fallot repair at young age: longitudinal follow-up up to 50 years after surgery. Int. J. Cardiol. 423, 133005. 10.1016/j.ijcard.2025.133005 39870118

[B41] Kelly R. G. (2024). “Molecular pathways and animal models of tetralogy of fallot and double outlet right ventricle,” in Congenital Heart Diseases: The Broken Heart: Clinical Features, Human Genetics and Molecular Pathways, 645–659. 10.1007/978-3-031-44087-8_37 38884739

[B42] Kerstjens-Frederikse W. S. Van De L. I. M. Vos Y. J. Verhagen J. Berger R. M. Lichtenbelt K. D. (2016). Cardiovascular malformations caused by NOTCH1 mutations do not keep left: data on 428 probands with left-sided CHD and their families. Genet. Med. 18 (9), 914–923. 10.1038/gim.2015.193 26820064

[B43] Kirby M. L. Gale T. F. Stewart D. E. (1983). Neural crest cells contribute to normal aorticopulmonary septation. Science. 220 (4601), 1059–1061. 10.1126/science.6844926 6844926

[B44] Klaus A. Müller M. Schulz H. Saga Y. Martin J. F. Birchmeier W. (2012). Wnt/β-catenin and Bmp signals control distinct sets of transcription factors in cardiac progenitor cells. Proc. Natl. Acad. Sci. 109 (27), 10921–10926. 10.1073/pnas.1121236109 22711842 PMC3390862

[B45] Koenig S. N. Bosse K. Majumdar U. Bonachea E. M. Radtke F. Garg V. (2016). Endothelial Notch1 is required for proper development of the semilunar valves and cardiac outflow tract. J. Am. Heart Assoc. 5 (4), e003075. 10.1161/JAHA.115.003075 27107132 PMC4843530

[B46] Kruszka P. Tanpaiboon P. Neas K. Crosby K. Berger S. I. Martinez A. F. (2017). Loss of function in ROBO1 is associated with tetralogy of fallot and septal defects. J. Medical Genetics 54 (12), 825–829. 10.1136/jmedgenet-2017-104611 28592524

[B47] Lamers W. H. Moorman A. F. (2002). Cardiac septation: a late contribution of the embryonic primary myocardium to heart morphogenesis. Circ. Res. 91 (2), 93–103. 10.1161/01.res.0000027135.63141.89 12142341

[B48] Lee Y.-H. Saint-Jeannet J.-P. (2011). Cardiac neural crest is dispensable for outflow tract septation in xenopus. Development 138 (10), 2025–2034. 10.1242/dev.061614 21490068 PMC3082305

[B49] Li Q. Pan H. Guan L. Su D. Ma X. (2012). CITED2 mutation links congenital heart defects to dysregulation of the cardiac gene VEGF and PITX2C expression. Biochem. Biophysical Research Communications 423 (4), 895–899. 10.1016/j.bbrc.2012.06.099 22735262

[B50] Li Y. Klena N. T. Gabriel G. C. Liu X. Kim A. J. Lemke K. (2015). Global genetic analysis in mice unveils central role for cilia in congenital heart disease. Nature 521 (7553), 520–524. 10.1038/nature14269 25807483 PMC4617540

[B51] Li D. Sinha T. Ajima R. Seo H.-S. Yamaguchi T. P. Wang J. (2016). Spatial regulation of cell cohesion by Wnt5a during second heart field progenitor deployment. Dev. Biology 412 (1), 18–31. 10.1016/j.ydbio.2016.02.017 26916252 PMC4814329

[B52] Li D. Angermeier A. Wang J. (2019). Planar cell polarity signaling regulates polarized second heart field morphogenesis to promote both arterial and venous pole septation. Development 146 (20), dev181719. 10.1242/dev.181719 31488563 PMC6826042

[B53] Lin Q. Schwarz J. Bucana C. Olson E N. (1997). Control of mouse cardiac morphogenesis and myogenesis by transcription factor MEF2C. Science 276 (5317), 1404–1407. 10.1126/science.276.5317.1404 9162005 PMC4437729

[B54] Liu Y. Feng Q. (2012). NOing the heart: role of nitric oxide synthase-3 in heart development. Differentiation 84 (1), 54–61. 10.1016/j.diff.2012.04.004 22579300

[B55] Loomes K. M. Taichman D. B. Glover C. L. Williams P. T. Markowitz J. E. Piccoli D. A. (2002). Characterization of Notch receptor expression in the developing mammalian heart and liver. Am. Journal Medical Genetics 112 (2), 181–189. 10.1002/ajmg.10592 12244553

[B56] Majumdar U. Yasuhara J. Garg V. (2021). *In vivo* and *in vitro* genetic models of congenital heart disease. Cold Spring Harb. Perspectives Biology 13 (4), a036764. 10.1101/cshperspect.a036764 31818859 PMC7307584

[B57] Matos-Nieves A. Manivannan S. Majumdar U. McBride K. L. White P. Garg V. (2021). A multi-omics approach using a mouse model of cardiac malformations for prioritization of human congenital heart disease contributing genes. Front. Cardiovascular Medicine 8, 683074. 10.3389/fcvm.2021.683074 34504875 PMC8421733

[B58] McElhinney D. B. Krantz I. D. Bason L. Piccoli D. A. Emerick K. M. Spinner N. B. (2002). Analysis of cardiovascular phenotype and genotype-phenotype correlation in individuals with a JAG1 mutation and/or Alagille syndrome. Circulation 106 (20), 2567–2574. 10.1161/01.cir.0000037221.45902.69 12427653

[B59] Medioni C. Bertrand N. Mesbah K. Hudry B. Dupays L. Wolstein O. (2010). Expression of slit and Robo genes in the developing mouse heart. Dev. Dyn. 239 (12), 3303–3311. 10.1002/dvdy.22449 20941780 PMC2996720

[B60] Mommersteeg M. T. Yeh M. L. Parnavelas J. G. Andrews W. D. (2015). Disrupted slit-robo signalling results in membranous ventricular septum defects and bicuspid aortic valves. Cardiovasc. Res. 106 (1), 55–66. 10.1093/cvr/cvv040 25691540 PMC4362403

[B61] Nagy I. I. Railo A. Rapila R. Hast T. Sormunen R. Tavi P. (2010). Wnt-11 signalling controls ventricular myocardium development by patterning N-cadherin and β-catenin expression. Cardiovasc. Research 85 (1), 100–109. 10.1093/cvr/cvp254 19622544

[B62] Neill C. A. Clark E. B. (1994). Tetralogy of fallot. The first 300 years. Tex Heart Inst. J. 21 (4), 272–279. 7888802 PMC325189

[B63] Page D. J. Miossec M. J. Williams S. G. Monaghan R. M. Fotiou E. Cordell H. J. (2019). Whole exome sequencing reveals the major genetic contributors to nonsyndromic tetralogy of fallot. Circulation Research 124 (4), 553–563. 10.1161/CIRCRESAHA.118.313250 30582441 PMC6377791

[B64] Pizzuti A. Sarkozy A. Newton A. L. Conti E. Flex E. Cristina Digilio M. (2003). Mutations of ZFPM2/FOG2 gene in sporadic cases of tetralogy of fallot. Hum. Mutation 22 (5), 372–377. 10.1002/humu.10261 14517948

[B65] Qiao X.-H. Wang F. Zhang X.-L. Huang R.-T. Xue S. Wang J. (2017). MEF2C loss-of-function mutation contributes to congenital heart defects. Int. Journal Medical Sciences 14 (11), 1143–1153. 10.7150/ijms.21353 29104469 PMC5666546

[B66] Rammeloo L. DeRuiter M. van Den Akker N. Wisse L. Gittenberger-de Groot A. (2015). Development of major aorto-pulmonary collateral arteries in vegf120/120 isoform mouse embryos with tetralogy of fallot. Pediatr. Cardiology 36 (1), 89–95. 10.1007/s00246-014-0969-4 25070391

[B67] Ramsbottom S. A. Sharma V. Rhee H. J. Eley L. Phillips H. M. Rigby H. F. (2014). Vangl2-regulated polarisation of second heart field-derived cells is required for outflow tract lengthening during cardiac development. PLoS Genetics 10 (12), e1004871. 10.1371/journal.pgen.1004871 25521757 PMC4270488

[B68] Rudloff S. Kemler R. (2012). Differential requirements for β-catenin during mouse development. Development 139 (20), 3711–3721. 10.1242/dev.085597 22991437

[B69] Sakata Y. Koibuchi N. Xiang F. Youngblood J. M. Kamei C. N. Chin M. T. (2006). The spectrum of cardiovascular anomalies in CHF1/Hey2 deficient mice reveals roles in endocardial cushion, myocardial and vascular maturation. J. Mol. Cell. Cardiol. 40 (2), 267–273. 10.1016/j.yjmcc.2005.09.006 16242143

[B70] Schleiffarth J. R. Person A. D. Martinsen B. J. Sukovich D. J. Neumann A. Baker C. V. (2007). Wnt5a is required for cardiac outflow tract septation in mice. Pediatr. Research 61 (4), 386–391. 10.1203/pdr.0b013e3180323810 17515859

[B71] Shi Y. Li Y. Wang Y. Zhu P. Chen Y. Wang H. (2020). BVES downregulation in non-syndromic tetralogy of fallot is associated with ventricular outflow tract stenosis. Sci. Rep. 10 (1), 14167. 10.1038/s41598-020-70806-4 32843646 PMC7447802

[B72] Sinha T. Li D. Théveniau-Ruissy M. Hutson M. R. Kelly R. G. Wang J. (2015). Loss of Wnt5a disrupts second heart field cell deployment and may contribute to OFT malformations in DiGeorge syndrome. Hum. Molecular Genetics 24 (6), 1704–1716. 10.1093/hmg/ddu584 25410658 PMC4381755

[B73] Smoak I. W. Byrd N. Abu-Issa R. Goddeeris M. Anderson R. Morris J. (2005). Sonic hedgehog is required for cardiac outflow tract and neural crest cell development. Dev. Biology 283 (2), 357–372. 10.1016/j.ydbio.2005.04.029 15936751

[B74] Song L. Li Y. Wang K. Zhou C. J. (2010). Cardiac neural crest and outflow tract defects in Lrp6 mutant mice. Dev. Dynamics 239 (1), 200–210. 10.1002/dvdy.22079 19705442

[B75] Stainier D. Y. Fouquet B. Chen J.-N. Warren K. S. Weinstein B. M. Meiler S. E. (1996). Mutations affecting the formation and function of the cardiovascular system in the zebrafish embryo. Development 123 (1), 285–292. 10.1242/dev.123.1.285 9007248

[B76] Starr J. P. (2010). Tetralogy of fallot: yesterday and today. World Journal Surgery 34 (4), 658–668. 10.1007/s00268-009-0296-8 20091166

[B77] Stefanovic S. Etchevers H. C. Zaffran S. (2021). Outflow tract formation—embryonic origins of conotruncal congenital heart disease. J. Cardiovasc. Dev. Dis. 8 (4), 42. 10.3390/jcdd8040042 33918884 PMC8069607

[B78] Stern C. D. (2005). The chick: a great model system becomes even greater. Dev. Cell 8 (1), 9–17. 10.1016/j.devcel.2004.11.018 15621526

[B79] Tang C. S. M. Mononen M. Lam W.-Y. Jin S. C. Zhuang X. Garcia-Barcelo M.-M. (2022). Sequencing of a Chinese tetralogy of fallot cohort reveals clustering mutations in myogenic heart progenitors. JCI Insight 7 (2), e152198. 10.1172/jci.insight.152198 34905512 PMC8855809

[B80] Tevosian S. G. Deconinck A. E. Tanaka M. Schinke M. Litovsky S. H. Izumo S. (2000). FOG-2, a cofactor for GATA transcription factors, is essential for heart morphogenesis and development of coronary vessels from epicardium. Cell 101 (7), 729–739. 10.1016/s0092-8674(00)80885-5 10892744

[B81] Timmerman L. A. Grego-Bessa J. Raya A. Bertran E. Perez-Pomares J. M. Diez J. (2004). Notch promotes epithelial-mesenchymal transition during cardiac development and oncogenic transformation. Genes Dev. 18 (1), 99–115. 10.1101/gad.276304 14701881 PMC314285

[B82] van den Akker N. M. Molin D. G. Peters P. P. Maas S. Wisse L. J. van Brempt R. (2007). Tetralogy of fallot and alterations in vascular endothelial growth factor-A signaling and notch signaling in mouse embryos solely expressing the VEGF120 isoform. Circulation Research 100 (6), 842–849. 10.1161/01.RES.0000261656.04773.39 17332426

[B83] van Vliet P. P. Lin L. Boogerd C. J. Martin J. F. Andelfinger G. Grossfeld P. D. (2017). Tissue specific requirements for WNT11 in developing outflow tract and dorsal mesenchymal protrusion. Dev. Biology 429 (1), 249–259. 10.1016/j.ydbio.2017.06.021 28669819 PMC5580348

[B84] Vitelli F. Morishima M. Taddei I. Lindsay E. A. Baldini A. (2002). Tbx1 mutation causes multiple cardiovascular defects and disrupts neural crest and cranial nerve migratory pathways. Hum. Molecular Genetics 11 (8), 915–922. 10.1093/hmg/11.8.915 11971873

[B85] Ward C. Stadt H. Hutson M. Kirby M. L. (2005). Ablation of the secondary heart field leads to tetralogy of fallot and pulmonary atresia. Dev. Biology 284 (1), 72–83. 10.1016/j.ydbio.2005.05.003 15950213

[B86] Waldo K. L. Hutson M. R. Stadt H. A. Zdanowicz M. Zdanowicz J. Kirby M. L. (2005). Cardiac neural crest is necessary for normal addition of the myocardium to the arterial pole from the secondary heart field. Dev. Biology 281 (1), 66–77. 10.1016/j.ydbio.2005.02.011 15848389

[B87] Wang H. Yong X. Gao Y. Li W. Chen Z. Liu Z. (2026). Integrative analysis of bulk and single-nucleus transcriptomes suggests proteostasis-and metabolism-related alterations in the right ventricular outflow tract of non-syndromic tetralogy of fallot. Funct. and Integr. Genomics 26 (1), 121. 10.1007/s10142-026-01898-w 42230411

[B88] Wu M. Li Y. He X. Shao X. Yang F. Zhao M. (2013). Mutational and functional analysis of the BVES gene coding region in Chinese patients with non-syndromic tetralogy of fallot. Int. Journal Molecular Medicine 31 (4), 899–903. 10.3892/ijmm.2013.1275 23403794

[B89] Xiang F. Sakata Y. Cui L. Youngblood J. M. Nakagami H. Liao J. K. (2006). Transcription factor CHF1/Hey2 suppresses cardiac hypertrophy through an inhibitory interaction with GATA4. Am. J. Physiology-Heart Circulatory Physiology 290 (5), H1997–H2006. 10.1152/ajpheart.01106.2005 16603706 PMC2692281

[B90] Xu M. Wu X. Li Y. Yang X. Hu J. Zheng M. (2014). CITED2 mutation and methylation in children with congenital heart disease. J. Biomed. Sci. 21 (1), 7. 10.1186/1423-0127-21-7 24456003 PMC3917535

[B91] Yamaguchi T. P. Bradley A. McMahon A. P. Jones S. (1999). A Wnt5a pathway underlies outgrowth of multiple structures in the vertebrate embryo. Development 126 (6), 1211–1223. 10.1242/dev.126.6.1211 10021340

[B92] Yelbuz T. M. Waldo K. L. Kumiski D. H. Stadt H. A. Wolfe R. R. Leatherbury L. (2002). Shortened outflow tract leads to altered cardiac looping after neural crest ablation. Circulation 106 (4), 504–510. 10.1161/01.cir.0000023044.44974.8a 12135953

[B93] Yin Z. Haynie J. Yang X. Han B. Kiatchoosakun S. Restivo J. (2002). The essential role of Cited2, a negative regulator for HIF-1α, in heart development and neurulation. Proc. Natl. Acad. Sci. 99 (16), 10488–10493. 10.1073/pnas.162371799 12149478 PMC124951

[B94] Zelzer E. McLean W. Ng Y.-S. Fukai N. Reginato A. M. Lovejoy S. (2002). Skeletal defects in VEGF120/120 mice reveal multiple roles for VEGF in skeletogenesis. Development 129 (8), 1893–1904. 10.1242/dev.129.8.1893 11934855

[B95] Zhang Z. Baldini A. (2008). *In vivo* response to high-resolution variation of Tbx1 mRNA dosage. Hum. Molecular Genetics 17 (1), 150–157. 10.1093/hmg/ddm291 17916582

[B96] Zhou W. Lin L. Majumdar A. Li X. Zhang X. Liu W. (2007). Modulation of morphogenesis by noncanonical Wnt signaling requires ATF/CREB family–mediated transcriptional activation of TGFβ2. Nat. Genetics 39 (10), 1225–1234. 10.1038/ng2112 17767158 PMC5578467

[B97] Zhou Y. Jiang T. Gao J. Zang J. Mo X. Yue S. (2025). Loss-of-function variants in ciliary genes confer high risk for tetralogy of fallot. Sci. Adv. 11 (41), eadt0836. 10.1126/sciadv.adt0836 41071877 PMC12513439

[B98] Zulibiya A. Wen J. Yu H. Chen X. Xu L. Ma X. (2023). Single-cell RNA sequencing reveals potential for endothelial-to-mesenchymal transition in tetralogy of fallot. Congenit. Heart Dis. 18 (6), 611–625. 10.32604/chd.2023.047689

